# Characterization and quantification of microplastics in marine sediments of the Colombian Caribbean

**DOI:** 10.7717/peerj.21127

**Published:** 2026-09-02

**Authors:** Luis Garzón-Rodríguez, Paulo Tigreros-Benavides, Jesús Ochoa-Mogollón, Gysseth Herrera-Villarraga, Camila Sarmiento-Sánchez, Paula Polania-Zenner, Gladys Rozo-Torres, Adolfo Sanjuan-Muñoz, Andrés Franco-Herrera

**Affiliations:** Área de Ciencias Biológicas y Ambientales, Facultad de Ciencias Naturales e Ingeniería, Universidad de Bogotá Jorge Tadeo Lozano, Santa Marta, Magdalena, Colombia

**Keywords:** Microplastic morphology, Microplastic composition, Magdalena River, Sinú River, Marine sediments

## Abstract

**Background:**

About eight million tons of plastic end up in the oceans every year, with negative impacts including effects on the marine biota, the accumulation of heavy metals and desorption of organic contaminants in marine sediments.

**Methods:**

With the aim of quantifying and characterizing the microplastics (MP) in the marine-coastal and lagoonal environments of two sectors of the Colombian Caribbean, influenced respectively by the Magdalena and Sinú rivers, sediment samples were collected during the 2021 rainy season and the 2022 dry season. The MP pieces (<5 mm) were extracted manually and classified by size (0.3–1.4 mm and 1.4–5.0 mm) and shape (fibers, films, foams and fragments). Moreover, from a total of 2,970 particles, a subset of 54 particles of more than 1.4 mm of both sectors was analyzed *via* Fourier-Transform Infrared (FTIR) spectrometer.

**Results:**

In both the environments, in Magdalena the highest concentrations of MP were found in the dry season (275.56 ± 221.12 MP/kg and 217.78 ± 126.84 MP/kg in lagoonal and marine-coastal, respectively) while in Sinú it was the rainy season when the highest values were reported (208.89 ± 137.56 MP/kg and 135.56 ± 71.45 MP/kg in lagoonal and marine-coastal, respectively). The dominant shape of MP was fibers and the predominant polymer was polypropylene. Although there was no significant effect of season (Permanova, *p* = 0.0058) nor environment (Permanova, *p* = 0.0741) there were differences between some in size (Permanova, *p* = 0.0001) which was noticeable due particles measuring 0.3–1.4 mm exceeded those measuring 1.4–5.0 mm by an order of magnitude. It was not found a clear relation between particle size and MP concentrations.

## Introduction

About eight million tons of plastic end up in the oceans every year and it is estimated that by 2050 it will exceed the biomass of marine fish (Zhao et al., 2022). Since plastics were first discovered, in the mid- to late 19th century, they have been incorporated into most aspects of daily life (Sivan, 2011; Wesch et al., 2017) and are recognized to represent an environmental problem, which has been extensively studied because of its artificial nature (Cole et al., 2011) and extremely slows rates of degradation (Barnes et al., 2009). Exposed to the elements, subject to the mechanical effects of wave action, and abrasion by sand, they can be broken down into fragments measuring less than five mm, a size below which they are classified as microplastics (MP) (Masura et al., 2015), which make up more than 90% of floating plastic rubbish (Eriksen et al., 2014). During the last four decades, an increased presence of MP has been recorded in all the world’s oceans, leading to elevated levels of environmental concern (Derraik, 2002; Thompson et al., 2004; Barnes et al., 2009).

MP have been categorized according to their origin. If they were designed and manufactured at this size for use in industry they are known as primary, while pieces that have been produced as a result of the fragmentation of larger pieces are considered secondary (Komyakova, Vince & Haward, 2020). They form a heterogenous group, varying in shape, size, color, density, chemical composition and other characteristics (Hidalgo-Ruz et al., 2012) that confer on them significant potential to pollute, given the ease with which they can be introduced into aquatic environments. The recognized effects of MP in marine environments include their ingestion and accumulation by organisms, the ease with which they can enter the circulatory and lymphatic systems (Wright & Kelly, 2021; Wang, Ge & Yu, 2020), deterioration of the digestive tracts of fish (Pedà et al., 2016) and the adsorption and concentration of poisonous materials such as heavy metals and organic contaminants (Wang, Ge & Yu, 2020) with potentially toxic effects that are capable of reaching the human population through the food web.

These particles have been found in deep water sediments in the world’s principal oceans (Van Cauwenberghe et al., 2013; Bergmann et al., 2017), at concentrations as high as those reported for sediments close to the coast (Zhang et al., 2020), demonstrating that they can accumulate on the ocean floor when they increase in weight as a result of colonization by microorganisms (Matsuguma et al., 2017), the formation of agglomerates (Peng et al., 2020) or simply because they are denser than the sea water and are distributed through the water column as a result of the action of currents (Clark et al., 2016).

The MP pollution in Colombia is primarily related to tourism, insufficient sewage and wastewater management, urban development and intense coastal socioeconomic activities besides and increasing plastic consumption (from 1,075 tons/day in 2010 to 3,424 tons/day in 2019) (Galindo et al., 2023). Consequently, some researches have been carried out in some marine environments such as beaches (Rangel-Buitrago et al., 2018; Acosta-Coley et al., 2019; Garcés-Ordóñez et al., 2020a; Garcés-Ordóñez et al., 2020b; Rangel-Buitrago et al., 2021; Fuentes, López & Puerta, 2025; González-Curbelo et al., 2025), sea water (Garcés-Ordóñez et al., 2021; Garcés-Ordóñez et al., 2022b; Vidal, Molina & Duque, 2021; Franco-Herrera et al., 2022; Garcés-Ordóñez et al., 2023; Tigreros-Benavides et al., 2024) and only three studies focused on the sediments like of Ciénaga Grande de Santa Marta (Garcés-Ordóñez et al., 2019; Garcés-Ordóñez et al., 2022a; Garcés-Ordóñez et al., 2022b) and Buenaventura Bay (Vásquez-Molano, Molina & Duque, 2021). While unlike the previous studies related to characterizing MP pollution, some others addressed the ecological and environmental impact of MP (Rusinque-Quintero, Montoya-Rojas & Moyano-Molano, 2022; Fuentes, López & Puerta, 2025) and microorganisms associated to these pollutants (Tigreros-Benavides et al., 2024).

Therefore, this article came to the research question. What is the level of MP pollution in the marine sediments of Colombian Caribbean and how it is linked to its marine-coastal environmental factors? Hence, the aim of this research was to describe MP pollution in in marine sediments under contrasting climatic conditions in two sectors determined by the two most important rivers of the of the Colombian Caribbean basin: the Magdalena and Sinú rivers, in order to establish a scientific basis with which to contribute to the implementation of policies for the management of plastic waste. To achieve this goal (1) concentration of MP was quantified and their morphological traits (size shape) were detailed, (2) polymers present in these particles were determined, and (3) the relationship between these pollutants and environmental factors (climatic periods, kind of environment and anthropic activities, and gran size) was detailed while expecting higher amounts of smaller particles in the environments of Magdalena.

## Materials & Methods

### Study area

**Figure 1:**
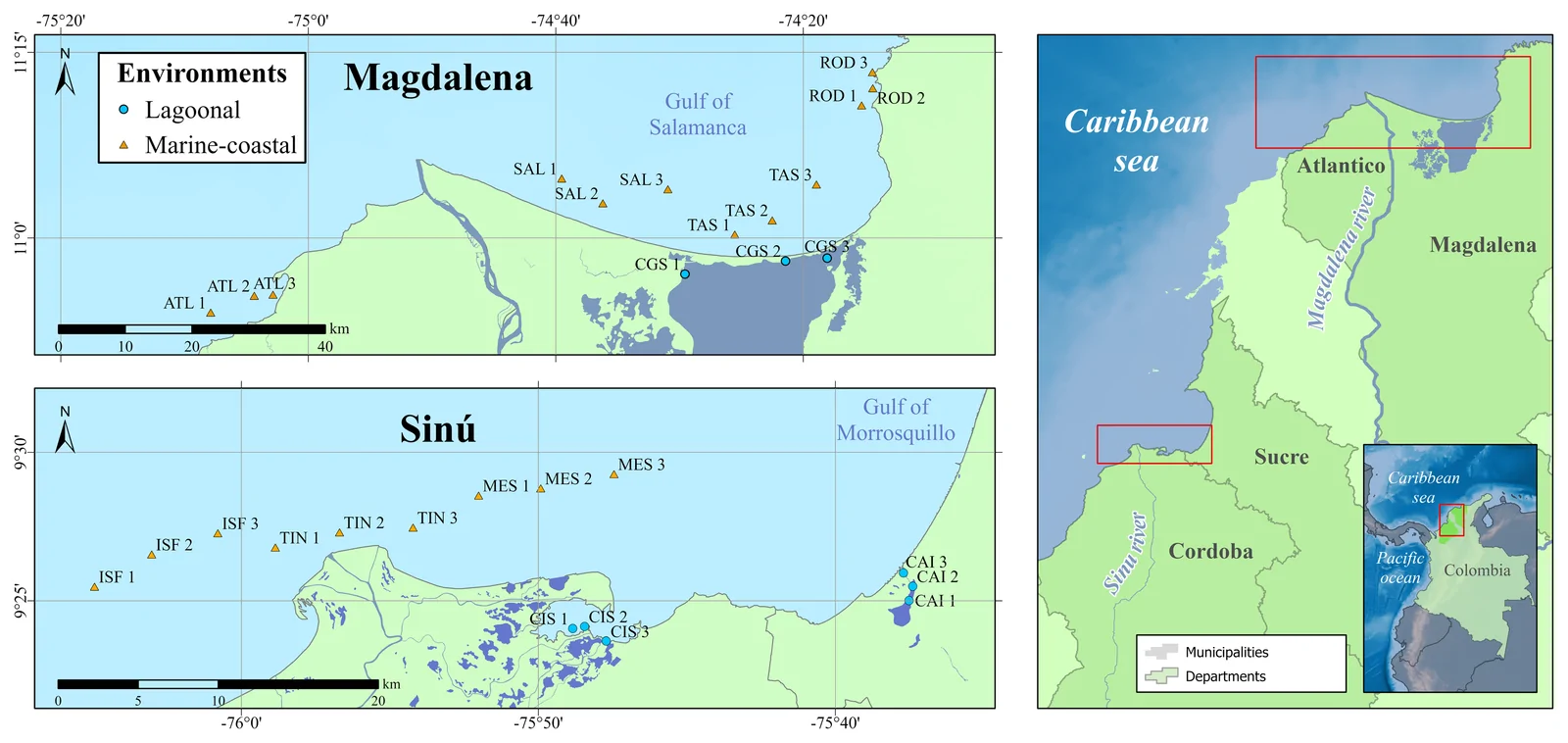
Locations and stations sampled of marine-coastal and lagoonal environments in both sectors. ATL, Atlántico; SAL, Salamanca; TAS, Tasajera; CGS, Ciénaga Grande de Santa Marta; ROD, El Rodadero; ISF, Isla Fuerte; TIN, Tinajones; MES, Mestizos; CIS, Cispatá bay; CAI, La Caimanera.

Sampling was carried out in two sectors influenced by the Magdalena and Sinú rivers. In both, two environments were chosen, the first referred hereafter as “marine-coastal” was composed by locations with three stations at a depth of less than 30 m each. The second environment was referred as “lagoonal” which was made of locations inside the coastal lagoons: Ciénaga Grande de Santa Marta (Magdalena), the Bahía de Cispatá and the Ciénaga de La Caimanera (Sinú) which were no deeper than 2 m (Fig. 1, Table S1).

In Magdalena, climatic periods are governed by the north-south migration of the Intertropical Convergence Zone, which causes the dry season (between December and April) and the rainy period (May to November). This is the overall climate pattern that characterizes Colombia’s Caribbean lowlands (Franco-Herrera, 2005). The environments in this sector are influenced by the principal river system of the region, the Magdalena, which contributes the greatest amount of suspended sediments (Restrepo-López et al., 2015). The lagoonal environment of this sector (complex of the Ciénaga Grande de Santa Marta) is known because it buffers the effects of floods and of the sediments carried by the rivers that descend from the western flanks of the Sierra Nevada de Santa Marta and by the different channels of the Magdalena (Restrepo & Kjerfve, 2000), while the marine-coastal environments is constantly exposed to highly populated areas such as Barranquilla, Santa Marta and El Rodadero, being the last two known as important tourist centers of the country (Guardiola, 2019). The environments in the Sinú sector are marked by the overall climatic patterns of the Colombian Caribbean coast, being influenced also by the deltaic plain of the Sinú River whose waters reach the lagoon systems in unimodal cycles (Ruiz-Ochoa, Bernal & Polanía, 2008). The lagoonal environment here is characterized by a network of oxbow lakes and fresh water lagoons and by a deltaic-estuarine zone, which includes the Bahía de Cispatá (mouth of the river) as well as a small coastal lagoon (La Caimanera), while the marine-coastal environment is composed of areas with non-enclosed waters located farther from the delta and are used as touristic hubs and fuel port terminals.

In Colombia, the urban development is linked to water bodies, however many human settlements lack a proper waste management, which means wastewater is not adequately treated and solid waste is poorly managed, that is, high amount of pollutants is discharged into rivers and marine waters (Garcés-Ordóñez et al., 2021). Therefore, the Magdalena and Sinú rivers generate contrasting oceanographic and socioeconomic environments. The Magdalena is 1,540 km long with a flow rate that reaches as much as 9,287 m^3^/s during the rainy season (Ricaurte-Villota & Bastidas-Salamanca, 2017), carrying with them sediments that constitute a central element of the morphodynamics of the coast and transfers significant amounts of nutrients to the Caribbean sea, transporting up to 142.6 Mt of suspended sediments every year, some 38% of the estimated total load for the country’s entire Caribbean coast (Restrepo-López et al., 2015). This river hosts major cities, runs through 18 departments and provides many ecosystem services to nearly 75% of Colombian population. In its lower basin, the Magdalena River runs between Atlántico and Magdalena departments, which collectively represent near 3 millions of inhabitants and 36.2% of their homes that lack municipal trash collection. While the principal activities carried out around this river are related to agriculture and livestock, maritime and port activities, tourism, industry and commerce (DANE-Departamento Administrativo Nacional De Estadística, 2018; Tejeda-Benítez et al., 2023; Centenaro et al., 2025).

On the other hand, the Sinú River is 415 km long and has an average flow rate of 383 m^3^/s. In contrast to the Magdalena, which discharges its sediments into the open ocean, the suspended sediments of the Sinú are deposited principally on the continental shelf, reaching an average annual total of 4.2 Mt, with the highest levels occurring in August and September (Tabares, Soltau & Diaz, 1996; Ercilla et al., 2002; Serrano, 2003). Moreover, since its origin the Sinú river goes through Córdoba and Sucre departments which host around one million of inhabitants which lack trash collection service in 56.3% of homes. Around 55% of the land area of Sinú basin is composed of agroecosystems related to multiple economic activities, particularly agriculture and cattle ranching. Furthermore, around its river mouth, many cities act as touristic hubs as well as centers of fishing activities and commerce (DANE-Departamento Administrativo Nacional De Estadística, 2018; Mandle et al., 2025).

### Sample collection

Samples were taken on two occasions during November 2021 (rainy season) and April 2022 (dry season). Three drags were conducted at every station using a stainless steel Van Veen grab sampler (0.05 m^2^) and the results integrated. The samples were stored in labeled boxes that were previously rinsed with filtered water (30 µm) and were maintained at ambient temperature until they were transferred to the laboratory. Exclusively for the granulometric analysis, three hundred grams of sediment were collected and stored in low density polyethylene bags.

### Separation and quantification of microplastics

Approximately 600 g of wet sediment from each sample were transferred to metal trays and dried in an oven at 60 °C for between 24 and 48 h (INVEMAR-Instituto De Investigaciones Marinas y Costeras & MADS-Ministerio De Ambiente y Desarrollo Sostenible, 2017). For each dried sample three fractions of 100 g were randomly selected from which MP were extracted independently once sediment was preprocessed as described below: the sediment particles were dispersed by adding 40 mL of an aqueous sodium hexametaphosphate solution (NaPO_3_)_6_ at 4.6 g/L and diluted at 1 L with microfiltered (GF/C 1.2 µm) deionized water, and then they were magnetically stirred for 15 min and left to settle for approximately 30 min (Eleftheriou & McIntyre, 2005). Consequently, 300 g of sediment were analyzed per station, that is, 4.5 kg from each sector per season. Considering the recommendations of GESAMP-Joint Group of Experts on the Science Aspects of Marine Environmental Protection (2019) the membrane sizes operationally-defined by the sieves available in Colombia (Tigreros-Benavides et al., 2024) were used to wet sieve the sediment using sizes 0.3 mm, 1.4 mm and five mm meshes along with microfiltered water in order to separate the samples into two classes referred hereafter as 0.3–1.4 mm and 1.4–5.0 mm sizes.

The identification and quantification of the MP was carried out using a stereoscope, individual pieces being extracted using stainless steel fine-tip tweezers, and the particles stored in 1.5 mL microcentrifuge tubes. The identity of particles of unclear nature was verified using the hot needle technique (De Witte et al., 2014). A complementary separation by shape was carried out, using the categorization proposed by Kovač-Viršek et al. (2016) and Rochman et al. (2019): fibers (or filaments), fragments, foam, films, granules and pellets.

### Granulometric analysis

The samples were dried in an oven at 90 °C for between 24 and 48 h. Samples that did not disaggregate were dissolved in 250 mL of deionized water with 10 mL de (NaPO_3_)_6_ to 10% (6.2 g/L) and subjected to magnetic stirring for 24 h. The dissolved sample was passed through a sieve tower graduated at 1,000, 500, 250, 180, 125, 90, 63 µm and the bottom pan. The fractions obtained were placed in Petri dishes, which had previously been weighed and labelled for drying in an oven at 90 °C for 24 h. When cool, the fractions were weighed using an Adventurer ARC117 scale. The dry non-conglomerated sediment samples were immediately passed through the screen tower and the weight of each fraction determined (Kenny & Sotheran, 2013).

### Identification of polymer type

Considering Bergmann, Gutow & Klages (2015), the chemical nature of the MP was determined by comparing the signals obtained using Attenuated Total Reflectance and Fourier-Transform Infrared (ATR-FTIR) using an Agilent Cary 630 FTIR spectrometer with a xenon flash lamp for the entire UV-Vis range: 190–1,100 nm. Signals were obtained for wavelengths from 4,000 cm^−1^ to 400 cm^−1^, proceeding to identification using Spectral Database System for Organic Compounds software, complemented by comparison of the signals obtained with existing commercial standards for polymers.

### Prevention of contamination

Cross-contamination by environmental particles was avoided by cleaning work surfaces and equipment with 15% sodium hypochlorite and 70% ethanol, by restricting the entry of personnel to laboratories and by refraining from using air conditioning. During particle extraction, the samples were handled using nitrile gloves and controls were conducted using blanks consisting of clean, open Petri dishes placed adjacently to the sample being observed. Once the analysis had been completed, it was reviewed in order to ascertain whether plastic pieces were present that might be environmental in origin, since they had not been found during initial inspection. The particles found were cleaned using microfiltered (GF/C 1.2 µm) deionized water.

### Data analysis

The concentration of MP was determined by dividing the amount of particles of each size in the mass of dry sediment analyzed for each station (0.3 kg) and thus expressing the data as MP/kg of dry sediment. The MP pollution analysis was performed *via* descriptive techniques using common statisticians and bar graph considering all the samples, and inferential analyses that employed Bray-Curtis similarity index, therefore only the samples from which MP were effectively identified (number of samples of 0.3–1.4 mm MP = 60 and number of samples of 1.4–5.0 mm MP = 58) as this index is undetermined between samples with double absences. The differences in the concentration by season (rainy or dry), environment (marine-coastal Magdalena, lagoonal Magdalena, marine-coastal Sinú, lagoonal Sinú) and size (0.3–1.4 mm and 1.4–5.0 mm) were determined using the similarity matrix and the PERMANOVA routine (Anderson, Gorley & Clarke, 2008), the latter using the statistical program PRIMER 7^®^ to consider the sum of type III squares and a full model of 9,999 permutations. Additionally, in an attempt to obtain a result in graphic form capable of presenting the results of the PERMANOVA, a re-sampling Bootstrap ordination analysis was carried out using randomizations to replace the concentrations and a multidimensional metric scaling (mMDS) ordination with regions of 95% (Clarke & Gorley, 2015). A descriptive analysis was carried out of the granulometry, verifying the changes in the distribution of granule size over space and time and evaluating their influence on the concentration of MP by developing distance-based linear models (DistLM), employing 9,999 permutations and using adjusted R^2^ (Anderson, Gorley & Clarke, 2008).

## Results

### Concentration of microplastics

MP were detected in 100% of the samples analyzed (30 in the rainy season and 30 in the dry season). Regardless the environment larger amounts of both particle sizes were found in Magdalena in the dry season, whereas m ore MP were reported in the rainy season in Sinú. The highest concentrations of Magdalena sector were found both in lagoonal (275 ± 221.12 MP/kg) and marine-coastal (217.78 ± 126.84 MP/kg) environments in the dry season but in the rainy season the amount of MP found in the lagoon system (87.78 ± 87.78 MP/kg) was exceeded by its counterpart (132.50 ± 90.59 MP/kg). In contrast, in Sinú despite the season, the lagoonal environment reached the greatest values (208.89 MP/kg and 178.33 MP/kg in rainy and dry season, respectively) compared to the marine-coastal area (135.56 ± 71.45 MP/g and 118.15 ± 48.91 MP/kg in rainy and dry seasons, respectively) (Table 1).

**Table 1:** Average ± standard deviation (minimum-maximum) of the concentration of microplastics (MP/kg) in each environment during the two climatic periods, by shape.

**Size**	**Environment**	**RS-FO**	**RS-FI**	**RS-FR**	**RS-FL**	**RS-MP**	**DS-FO**	**DS-FI**	**DS-FR**	**DS-FL**	**DS-MP**
**0.3–1.4 mm**	**MAG-MC**	1.39 ± 2.23 (0.00–6.67)	113.06 ± 87.46 (23.33–323.33)	–	1.67 ± 2.25 (0.00–6.67)	116.11 ± 90.82 (23.33–336.67)	0.83 ± 1.51 (0.00–3.33)	188.89 ± 111.04 (33.33–426.67)	0.28 ± 0.96 (0.00–3.33)	4.17 ± 5.15 (0.00–16.67)	194.17 ± 114.05 (36.67–443.33)
	**MAG-LG**	1.11 ± 1.92 (0.00–3.33)	67.78 ± 53.99 (33.33–130.00)	–	2.22 ± 3.85 (0.00–6.67)	71.11 ± 59.75 (33.33–140.00)	3.33 ± 3.33 (0.00–6.67)	245.56 ± 201.14 (110.00–476.67)	–	10.00 ± 11.55 (3.33–23.33)	258.89 ± 209.61 (120.00–500.00)
	**SIN-MC**	0.74 ± 1.47 (0.00–3.33)	115.56 ± 62.05 (50.00–226.67)	0.74 ± 1.47 (0.00–3.33)	5.19 ± 7.09 (0.00–16.67)	122.22 ± 63.36 (50.00–243.33)	1.11 ± 3.33 (0.00–10.00)	104.44 ± 47.08 (66.67–190.00)	–	6.30 ± 8.41 (0.00–20.00) 133.5%	111.85 ± 47.67 (66.67–190.00) 42.6%
	**SIN-LG**	3.89 ± 3.28 (0.00–6.67)	176.11 ± 114.88 (40.00–340.00)	2.78 ± 4.43 (0.00–10.00)	5.56 ± 10.68 (0.00–26.67)	188.33 ± 123.32 (40.00–346.67)	1.11 ± 1.72 (0.00–3.33)	165.56 ± 38.57 (110.00–206.67)	0.56 ± 1.36 (0.00–3.33)	5.00 ± 5.06 (0.00–13.33)	172.22 ± 41.72 (110.00–213.33)
**1.4-5.0 mm**	**MAG-MC**	–	13.89 ± 12.46 (0.00–33.33)	–	2.50 ± 5.34 (0.00–16.67)	16.39 ± 15.86 (0.00–43.33)	–	22.50 ± 16.94 (3.33–53.33)	0.28 ± 0.96 (0.00–3.33)	0.83 ± 2.07 (0.00–6.67)	23.61 ± 17.32 (3.33–53.33)
	**MAG-LG**	–	14.44 ± 7.70 (10.00–23.33)	2.22 ± 3.85 (0.00–6.67)	–	16.67 ± 11.55 (10.00–30.00)	1.11 ± 1.92 (0.00–3.33)	13.33 ± 5.77 (10.00–20.00)	–	2.22 ± 3.85 (0.00–6.67)	16.67 ± 11.55 (10.00–30.00)
	**SIN-MC**	0.37 ± 1.11 (0.00–3.33)	12.59 ± 9.09 (3.33–26.67)	–	0.37 ± 1.11 (0.00–3.33)	13.33 ± 10.14 (3.33–30.00)	–	5.56 ± 5.00 (0.00–16.67)	0.37 ± 1.11 (0.00–3.33)	0.37 ± 1.11 (0.00–3.33)	6.30 ± 4.23 (3.33–16.67)
	**SIN-LG**	2.78 ± 4.43 (0.00–10.00)	17.78 ± 13.11 (3.33–36.67)	–	–	20.56 ± 15.69 (3.33–46.67)	0.56 ± 1.36 (0.00–3.33)	4.44 ± 1.72 (3.33–6.67)	–	1.11 ± 1.72 (0.00–3.33)	6.11 ± 2.51 (3.33–10.00)
**Total**	**MAG-MC**	1.39 ± 2.23 (0.00–6.67)	126.94 ± 85.50 (36.67–326.67)	–	4.17 ± 5.88 (0.00–20.00)	132.50 ± 90.59 (36.67–340.00)	0.83 ± 1.51 (0.00–3.33)	211.39 ± 122.82 (46.67–470.00)	0.56 ± 1.92 (0.00–6.67)	5.00 ± 7.04 (0.00–23.33)	217.78 ± 126.84 (50.00–493.33)
	**MAG-LG**	1.11 ± 1.92 (0.00–3.33)	82.22 ± 51.03 (43.33–140.00)	2.22 ± 3.85 (0.00–6.67)	2.22 ± 3.85 (0.00–6.67)	87.78 ± 55.51 (43.33–150.00)	4.44 ± 1.92 (3.33–6.67)	258.89 ± 206.89 (120.00–496.67)	–	12.22 ± 15.40 (3.33–30.00)	275.56 ± 221.12 (130.00–530.00)
	**SIN-MC**	1.11 ± 2.36 (0.00–6.67)	128.15 ± 68.94 (53.33–253.33)	0.74 ± 1.47 (0.00–3.33)	5.56 ± 7.82 (0.00–20.00)	135.56 ± 71.45 (53.33–273.33)	1.11 ± 3.33 (0.00–10.00)	110.00 ± 47.26 (70.00–190.00)	0.37 ± 1.11 (0.00–3.33)	6.67 ± 8.16 (0.00–20.00)	118.15 ± 48.91 (70.00–193.33)
	**SIN-LG**	6.67 ± 6.32 (0.00–16.67)	193.89 ± 126.23 (46.67–376.67)	2.78 ± 4.43 (0.00–10.00)	5.56 ± 10.68 (0.00–26.67)	208.89 ± 137.56 (46.67–393.33)	1.67 ± 2.79 (0.00–6.67)	170.00 ± 38.93 (113.33–210.00)	0.56 ± 1.36 (0.00–3.33)	6.11 ± 5.74 (0.00–16.67)	178.33 ± 42.73 (113.33–220.00)

**Notes.**

RS Rainy season DS dry season FO foams FI fibers FR fragments FL films, and MP

**Figure 2:**
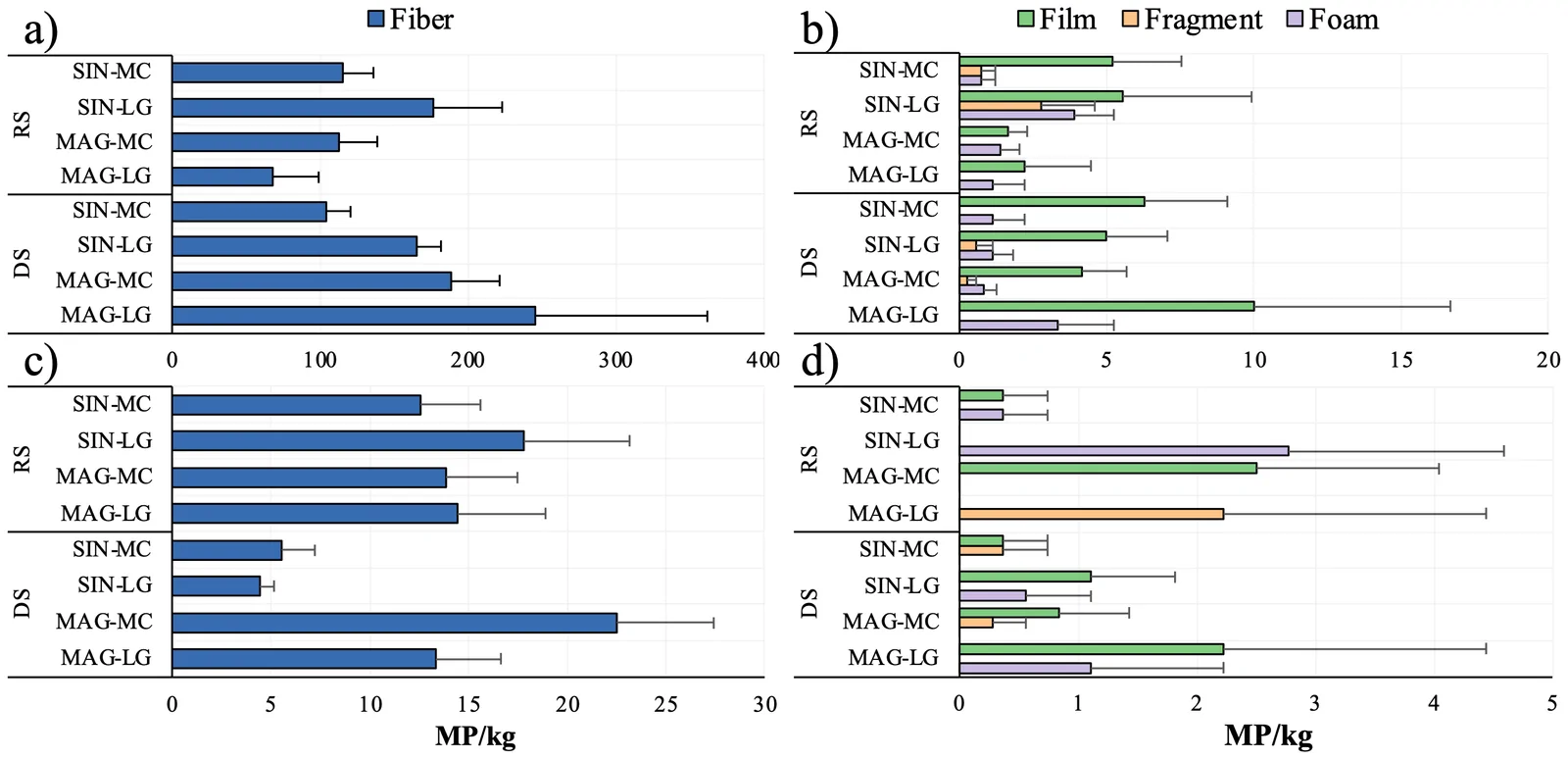
Concentration (MP/kg) of 0.3–1.4 mm (A, B) and 1.4–5.0 mm (C, D) of the shapes of microplastics. Marine-coastal (MC) and lagoonal (LG) environments of Magdalena (MAG) and Sinú (SIN) sectors during the rainy (RS) and dry seasons (DS).

Moreover, the concentrations of 0.3–1.4 mm MP were greater by an order of magnitude than those of 1.4–5.0 mm (Fig. 2). Regarding the latter size, the environments of Magdalena were more polluted than those of the Sinú sector. The highest concentrations of the dry and rainy seasons were found respectively in the marine-coastal environments of Magdalena (23.6 ± 17.3 MP/kg) and in the lagoonal environment of Sinú (20.6 ± 15.7 MP/kg). On the contrary, fewer particles were detected in the Sinú sector, reaching the lowest value in the lagoonal environment in the dry season (6.1 ± 2.5 MP/kg). In the case of 0.3–1.4 mm particles, in the dry season higher concentrations were reported in both marine-coastal (258.9 ± 209.6 MP7 kg) and lagoonal (194.2 ± 114.1 MP/kg) systems of Magdalena, while in the rainy season these reached lower values respect Sinú ecosystems (lagoonal: 188.3 ± 123.2 and marine-coastal: 122.2 ± 63.4 MP/kg). The dominant type in all the environments, and in both climatic seasons, for the two sizes, was fibers (95.1%), followed by films (3.4%), foams (1.1%) and fragments (0.4%); no primary MP, such as granules or pellets, were found (Fig. 2).

### Granulometry

**Figure 3:**
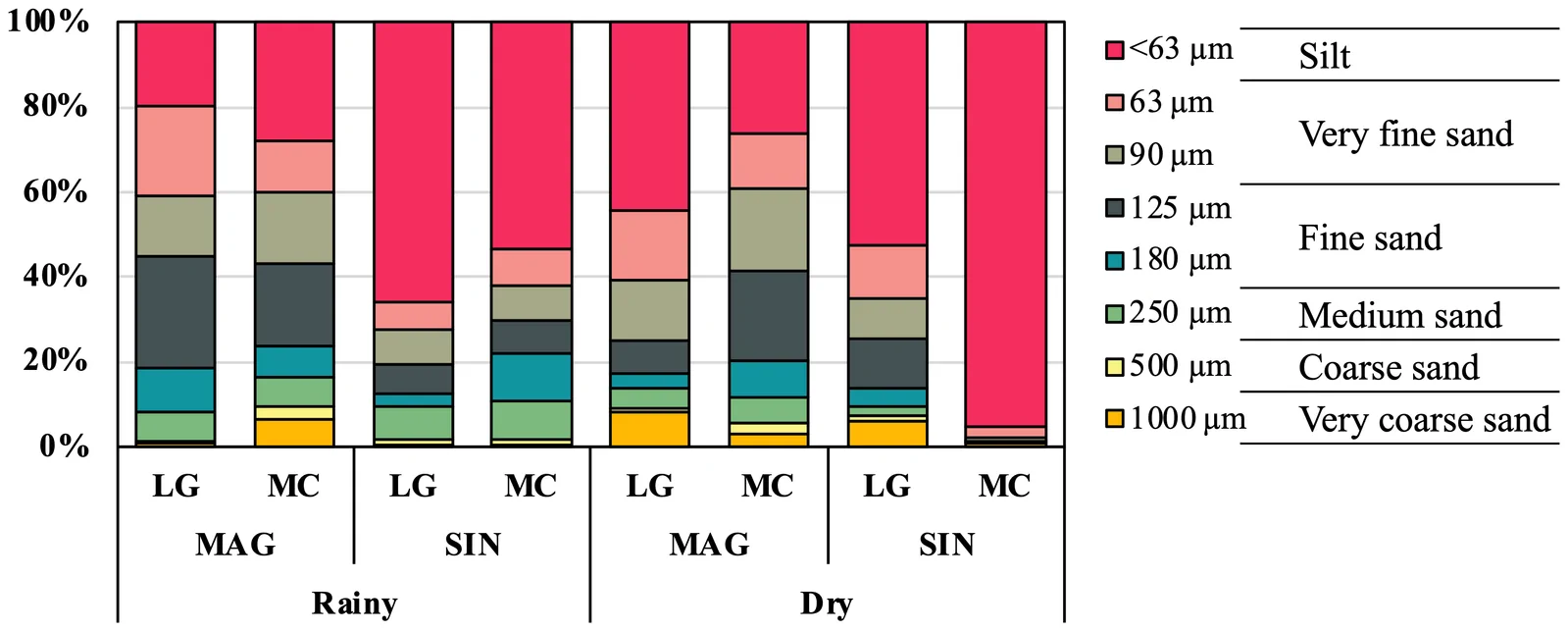
Relative abundance of grain size in the environments during both climatic periods. MAG, Magdalena sector; and SIN, Sinú sector; LG, Lagoonal; MC, Marine-coastal.

Silts dominated in all the environments of Sinú during both climatic periods, reaching up to 65.9 ± 19.5% in the rainy season and 28.1 ± 30.9% during the dry period. In Magdalena, this tendency was similar but in the lagoonal environment in the rainy season when fine sand (125 µm) was more abundant, however in the period the marine-coastal systems achieved the highest amount of silts (95.1 ± 5.9%) (Fig. 3). Coarse and very coarse sands (500 µm and 1,000 µm respectively) were less well represented, with a maximum value of coarse sand in the dry season in the marine-coastal environments of Sinú (3.4 ± 3.2%) and Magdalena (2.3 ± 5.0%).

The DistLM indicated that individually all of the grain sizes has low, non-significant, explanatory power concerning the concentration of MP for all the environments and both for 0.3–1.4 mm and 1.4–5.0 mm particles (Table S2). Regarding the sequential tests (Table S3), despite the grain size explained in a higher percentage the MP pollution of lagoonal environments than marine-coastal systems, the results were non-significant, therefore was not possible to confirm that grain size played a determining factor in the concentration of MP. However, when considering grains from silt (<63 µm) to coarse sand (500 µm) a 44.81% of 1.4–5.0 mm MP pollution in the lagoonal environments was significantly explained (pseudo-F = 4.96, *p* = 0.0302).

### Distribution of microplastics

**Figure 4:**
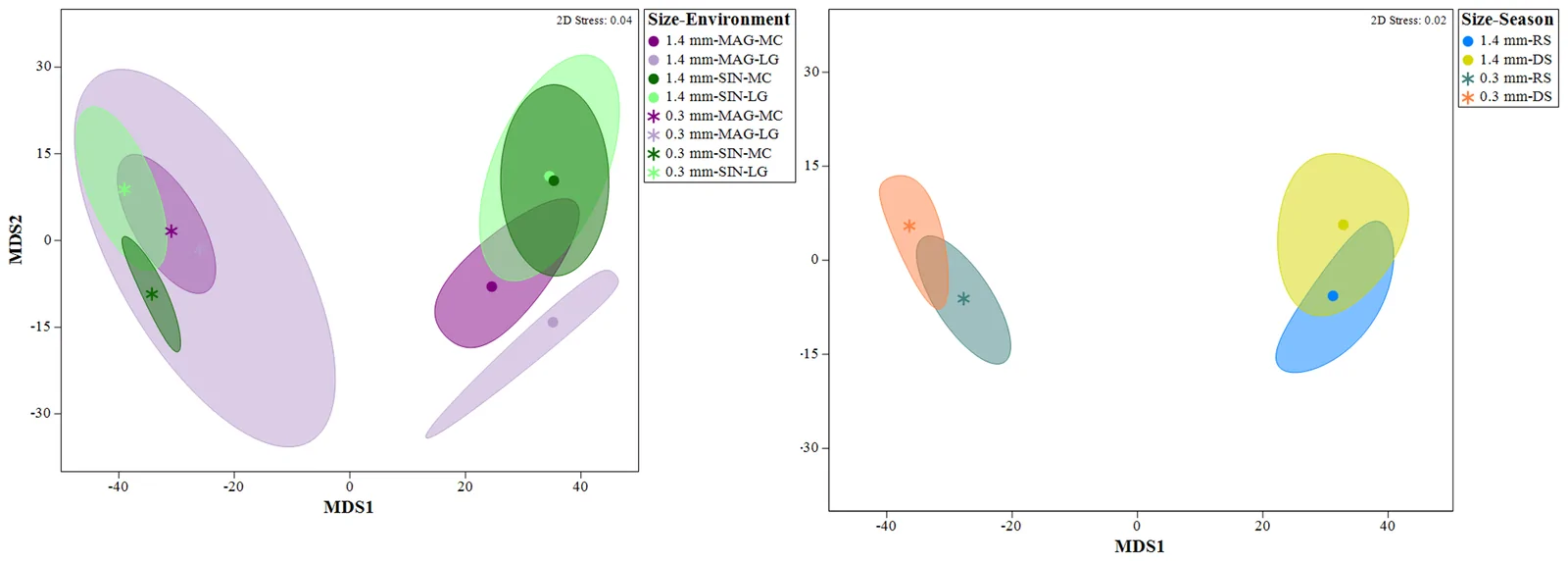
MMDS of bootstrap averages calculated from Bray-Curtis similarity matrix of MP concentrations of both environments, seasons and sizes. The positions of symbols represent the position of centroids per bootstrap, with shaded areas the 95% confidence intervals. MAG, Magdalena sector; and SIN, Sinú sector; LG, Lagoonal; MC, Marine-coastal.

As a result of the analysis, it was possible identify a segregation by size (Fig. 4), which was highly significant (Permanova, pseudo-F = 84.26, *p* = 0.001) and evident in all the environments and in both climatic periods, that is, 0.3–1.4 mm MP were significantly more abundant than 1.4–5.0 mm MP in all the cases (Fig. 2). In contrast, the environment factor alone wasn’t significant, but its interaction with particle size unveiled that only the marine-coastal environment of Magdalena exceeded the amount of 1.4–5.0 mm MP of its counterpart in Sinú (Permanova, pseudo-t = 1.67, *p* = 0.0443) as well as the lagoonal environment of this sector (Permanova, pseudo-t = 1.76, *p* = 0.0478). Moreover, when considering 0.3–1.4 mm MP the concentration of particles differed significantly between both environments in Sinú (Permanova, pseudo-t = 2.09, *p* = 0.0301) (Table S4).

When analyzing the distribution of MP of the marine-coastal environments of Magdalena and Sinú, it was found that their highest concentrations in both seasons were due to ROD seasons (RS: 23.8 ± 123.9 MP/kg, DS: 354.4 ± 155.4 MP/kg) and TIN (RS: 164.4 ± 98.0 MP/kg, DS: 158.9 ± 56.8 MP/kg), respectively. Indeed, ROD reached the highest values of the marine-coastal environment regardless the season. On the contrary, the lowest values of this environment in Magdalena and Sinú were found consistently in TAS (RS: 66.7 ± 36.1 MP/kg, DS: 147.8 ± 89.8 MP/kg) and ISF (RS: 96.7 MP/kg, DS: 78.9 ± 7.7 MP/kg), respectively. Furthermore, the distribution of MP within lagoonal environment showed that the highest values were reported in CGS only during dry season (275.6 ± 221.1 MP/kg), whereas in rainy season this locality reached the lowest concentrations (87.8 ± 55.5 MP/kg) in relation to CAI (316.7 ± 90.6 MP/kg) and CIS (194.4 ± 41.4 MP/kg) during the same period. Moreover, the latter locality the only one where there were more particles during the dry season, differing from the trend of Sinú (Table S5).

### Analysis of polymers

**Figure 5:**
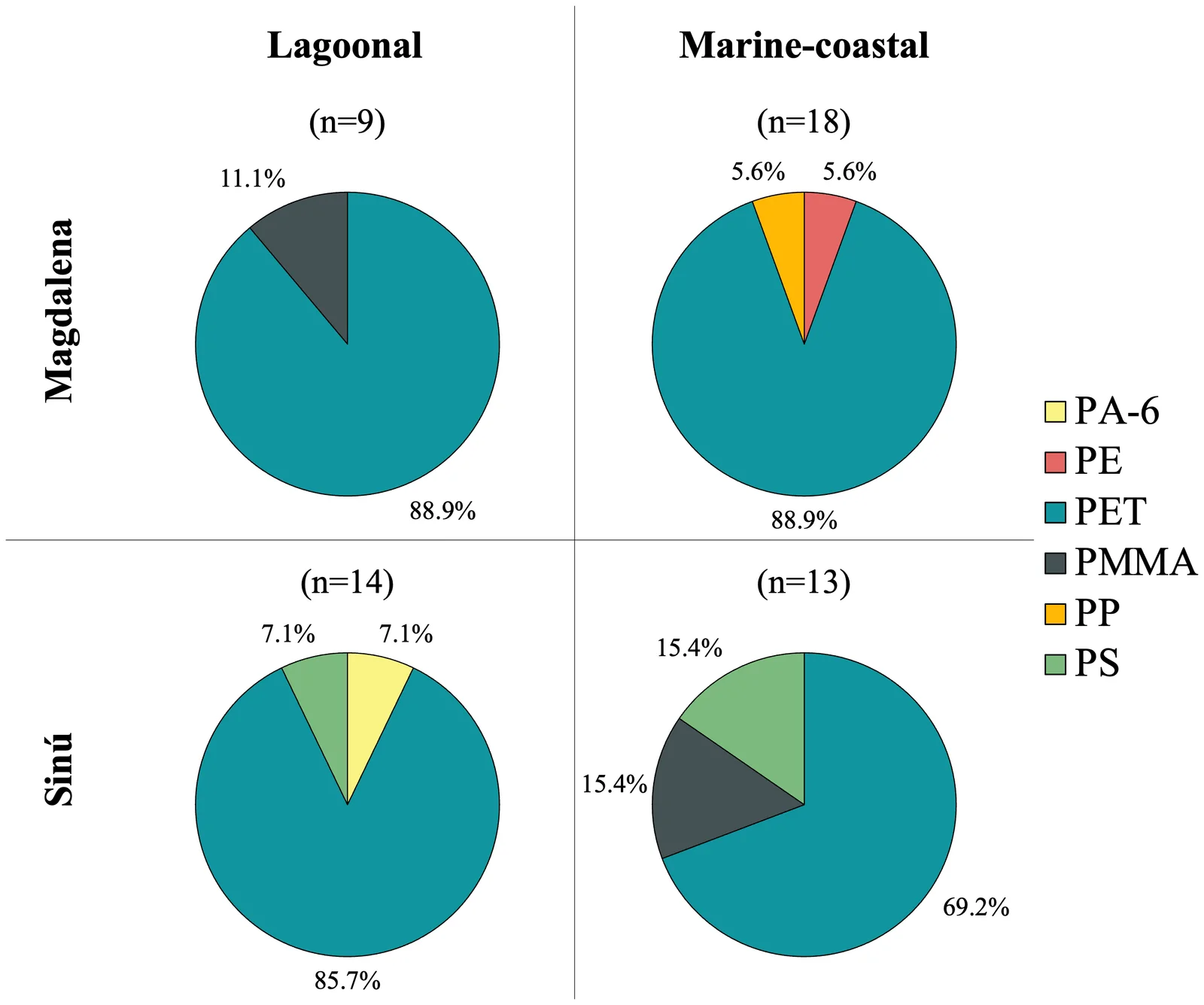
Relative abundance of polymers derived from 1.4–5.0 mm microplastics in both sectors. PET, polyethylene terephthalate; PMMA, poly(methyl methacrylate); PP, polypropylene; PE, polyethylene; PA-6, polyamide and PS, polystyrene.

Spectrums were obtained only for 1.4–5.0 mm MP, which were of sufficient size to detected by the spectrometer. Fifty-four positive results were recorded, corresponding to six polymers (Fig. S1), among which polyethylene terephthalate (PET) was the most abundant in all of the environments. In addition, some polymers were found exclusively in some locations, such as polyethylene (PE) in marine-coastal Magdalena, polyamide (PA6) and polystyrene (PS) in lagoonal Sinú and polypropylene was only reported only in the marine-coastal environment (Fig. 5).

## Discussion

### Concentration of MP

The presence of MP in 100% of the samples confirms the ubiquity of these particles in the sediments, consistent with the tendency reported for beaches, surface waters (Rangel-Buitrago et al., 2018; Acosta-Coley et al., 2019; Garcés-Ordóñez et al., 2020b; Rangel-Buitrago et al., 2021; Arboleda, Molina & Duque, 2024; Tigreros-Benavides et al., 2024) and sediments of coastal lagoons in the Colombian Caribbean (Garcés-Ordóñez et al., 2019; Garcés-Ordóñez et al., 2022b). The dominance of secondary MP, associated with industrial waste and cosmetic use indicate the possible sources of larger plastic items (packaging, bags, textile products and nets) that are degraded and fragmented as a result of wave action, UV radiation and sand abrasion (Thompson et al., 2004; Mathalon & Hill, 2014; Nor & Obbard, 2014; Fischer et al., 2015; Reed et al., 2018). These plastics have their origin in urban areas with inadequate sanitation systems, their preponderant contribution being magnified by their proximity to sources such as rivers and/or industry. In addition, the large presence of fibers indicates the significant role played by domestic activities associated with the washing of clothes, given that a single garment may render 1,900 fibers per wash (Browne et al., 2011). The deficiencies in the purification and treatment of residual waters in urban settlements leads to a large number of contaminants being released into rivers and ending up transported to marine-coastal environments. This transport may increase during periods of tourist activity, which might explain the higher concentrations in ROD as this location is in the city of Santa Marta and has been recognized for its tourist potential (Guardiola, 2019); it has experienced a 102.46% increase in domestic and international tourist arrivals in January 2022 compared to the same month in 2021 (MINCIT-Ministerio De Comercio e Industria y Turismo, 2022).

Although that there were significant differences between the climatic periods with higher concentrations in rainy season in Sinú environments, in Magdalena, the dry season seemed to increase MP pollution in both environments. The latter result contrasts with the significance of seasonal change found by Vásquez-Molano, Molina & Duque (2021) for Buenaventura Bay in Colombia and by Borges, Dzul & von Osten (2019) for the Gulf of México, both of whom reported that greater quantities of MP were deposited in sediments during the rainy season, possibly as a result of higher levels of flushing of macro and/or microplastics caused by increased runoff, which transport residues deposited on beaches and in streams and rivers, which are subsequently distributed by the action of waves, currents and winds (Baptista et al., 2019a; Borges, Dzul & Von Osten, 2019).

The relation between the concentration of MP and climatic period, which is evident in surface waters, is not necessarily mirrored in sediments, a finding that suggests that different factors affect their distribution at the sea surface levels and in sediments (Ronda et al., 2019). Hydrological processes are the principal causes of the difference between the spatial distribution of MP in both substrates, reflecting a spatial pattern in the sediments caused by constant accumulation under the long-term influence of human activities and annual mean currents, which, in surface waters, are dominated by short-term emissions and monthly mean currents (Teng et al., 2020). Therefore, even though seasonal changes affect MP pollution in sediments, it doesn’t involve uniform changes in magnitude nor direction, that is, the effects can be modulated by the environment, proximity to pollution sources, hydrodynamics, sediment characteristics and human activities (Díaz-Jaramillo, Islas & Gonzalez, 2021; Amenabar et al., 2024; Ruiz-Fernández et al., 2024).

It is important to consider the residence time of particles in the geomorphological unit under examination (McEachern et al., 2019). For example, in enclosed or semi-enclosed bays and/or estuarine systems the greater residence time permits epiplastic microbial communities to become established, contributing to the sedimentation of previously floating plastics (Andrady, 2011; Zettler, Mincer & Amaral-Zettler, 2013). In contrast, in open bays the high velocity of the currents is an obstacle to the settling of MP (Sui et al., 2020). This is consistent with the high concentrations of MP found in lagoon systems such as the ones of Magdalena and Sinú during rainy season (275.6 ± 221.1 MP/kg and 178.3 ± 42.7 MP/kg, respectively). Moreover, specific attributes of coastal lagoons might be related to different MP pollution dynamics, that is the case of CGS: despite both are relatively near to each other, CAI exhibits the traits a choked lagoon and therefore has less water exchange than other types (*e.g.*, leaky lagoons or restricted lagoons such as Cispatá and CGS, respectively), and its closer to human settlements, explaining the higher and more diverse MP pollution (Garcés-Ordóñez et al., 2022a; Garcés-Ordóñez et al., 2022b). However, sediments characteristics and hydrodynamic processes can homogenize MP distribution across different coastal environments and consequently the particles could be redistributed in sediments regardless of whether the environment, as a consequence sedimentation dynamic and mixing events can mask spatial differences in MP accumulation (Díaz-Jaramillo, Islas & Gonzalez, 2021; Garcés-Ordóñez et al., 2022a; Garcés-Ordóñez et al., 2022b) as evidenced in this study.

MP of 0.3–1.4 mm accounted for around 90% of the total in both the environments of Magdalena and Sinú. The concentrations of MP of this size significatively exceeded those of 1.4–5.0 mm by an order of magnitude. While the range of materials considered MP may include particles that measure as much as 5.0 mm, authors such as Wang et al. (2019), Li & Sun (2020) and Zhang et al. (2020) state that particles smaller than one mm dominate, as these are the ones that seem to prevail in marine sediments, with the result that the problem is more serious than is often argued. Cózar et al. (2014) argue that the smallest particles are removed from the water column and accumulated in the marine sediments by processes of colonization by microorganisms and the formation agglomerates that increase their density, reducing their floatability (Morét-Ferguson et al., 2010; Matsuguma et al., 2017; Peng et al., 2020) and promoting settling on the seabed or sinking, as marine snow (Galloway, Cole & Lewis, 2017). Once deposited in the seabed, the MP by themselves could directly and indirectly affect element cycling by increasing the carbon storage in sediment due their carbon-rich chemical structure and therefore changing the sediment carbon fixation and emission. In addition, the plastic breakdown through photodegradation and biodegradation leads to the growth and alter the enzyme activities of microbes related to carbon, nitrogen, phosphorous and sulfur cycling as well as they can inhibit the growth and activities of benthic organisms such as bioturbation of benthic fauna and so, influencing the redox micro-environment of sediment (Li et al., 2023). Moreover, as suggested by Rochman et al. (2019), the MP are considered as a suite of contaminants due they are vectors of heavy metals, polycyclic aromatic hydrocarbons, polychlorinated biphenyls and groups of microorganisms that might act as pathogens known as plastisphere (Amaral-Zettler, Zettler & Mincer, 2020).

### Comparisons within the Latin American and Greater Caribbean region

**Figure 6:**
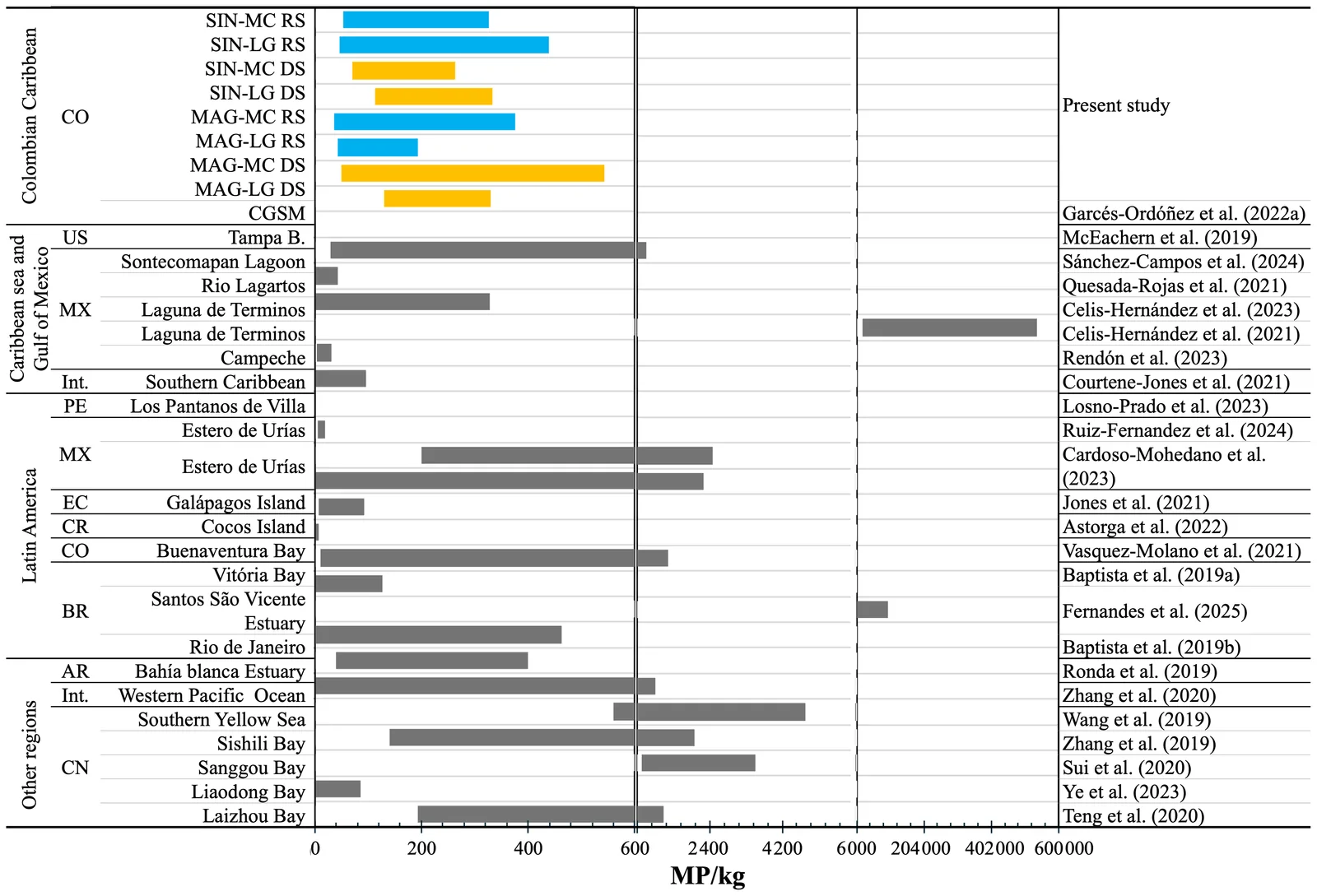
Concentrations of microplastics (MP/kg) in marine sediments reported in different studies. SIN, Sinú; MAG, Magdalena; MC, Marine-coastal environment; LG, Lagoonal environment; RS, Rainy season; DS, Dry season; CO, Colombia; US, United States of America; MX, Mexico; PE, Peru; EC, Ecuador; CR, Costa Rica; BR, Brazil; AR, Argentina; CN, China; Int., International. The data used for this figure is fully described in Table S6.

Latin America and the Caribbean are not alien to the global problem of a lack of standardized methodologies—a situation that affects research results as described by Pacheco et al. (2025). In this regard, Wang et al. (2019) mention that the depth of the sediment layer in which the samples are collected influences results, since the concentration of MP decreases significantly with depth, being at its greatest in the top five cm. To this should be added the absence of protocols covering the preparation of samples and methods, and the instrumentation used in identification and quantification (Hidalgo-Ruz et al., 2012), which lead to different results that make comparisons with other studies harder. One of these discrepancies consists in the fact the measurements are expressed in terms of concentration, which may be reported as the number of particles (also expressed as items or MP) or by area (Borges, Dzul & von Osten, 2019; Ramírez-Álvarez et al., 2019), while other studies express it in terms of weight (Baptista et al., 2019a; Baptista et al., 2019b; Ronda et al., 2019; Courtene-Jones et al., 2021; Jones et al., 2021; Garcés-Ordóñez et al., 2022b), as in this research. In the case of the Colombian Caribbean, only one study, of the benthic sediments of the Ciénaga Grande de Santa Marta exists, in which concentrations were reported to be lower than those recorded in this study (Fig. 6, Table S6).

Comparisons with studies conducted in the bays or coastal lagoons in the Caribbean (which report weight concentration) show similar levels in Rio Lagartos, Mexico (Quesada-Rojas, Enriquez & Valle-Levinson, 2021) and lower concentrations in Sontecomapan, Mexico (Sánchez-Campos et al., 2024) and in Laguna de Terminos, Mexico (Celis-Hernández et al., 2021), whereas a highly developed region as Tampa Bay, USA showed more MP in its sediments (McEachern et al., 2019). Despite being a protected area unlike Tampa Bay, the Laguna de Terminos reached more MP than the present study, in fact it proved to be 100 times more polluted than other areas (Celis-Hernández et al., 2023). When contrasting with studies carried out in marine-coastal systems of southern Gulf of Mexico, MP abundance was related to the proximity of touristic activities (hotels) and residential populations, where the plastic waste tends to be thrown away on the streets to be carried to the sea during rainy periods in coastal sampling sites, reaching higher levels than the present study (Borges, Dzul & von Osten, 2019) which is represented in this study by some areas such as marine-coastal environments of this study, particularly in El Rodadero (Table S1). Nevertheless, MP pollution seemed to decrease as sampling sites are deeper and more distant from the emission sources (Rendón-von Osten et al., 2023), obtaining lower values than in the sampled areas of Colombian Caribbean of this research. Moreover, when compared to Courtene-Jones et al. (2021), this study reached higher levels of MP compared to the southern Caribbean where, according to Langranian modelling, most of the marine plastic pollution had local sources (Panama, Aruba, Bonaire and Colombia) but in Antigua whose MP came from the Atlantic Ocean and appeared to travel by ocean currents that flow into the Caribbean.

By broadening the scope to other marine regions of Latin America it was found that in a Pacific estuarine ecosystem that has been highly intervened because of the most important seaport of Colombia, an increase of 84.4% of MP abundance was detected between 2015 and 2019 reaching similar levels (11–1,354 particles/kg) to those of lagoonal estuarine environments of this study (43.3–530 MP/kg) which is related to a poor environmental management of the city which major economic activity depends on its harbor (Vásquez-Molano, Molina & Duque, 2021). Similarly, higher levels of MP pollution were found in the Estero de Urias, a marine-coastal system of the Pacific coast of Mexico where tidal and wind-induced currents caused and homogenization process of MP concentration through the area (Cardoso-Mohedano et al., 2023; Ruiz-Fernández et al., 2024) as evidenced among most of the non-estuarine locations of this study which concentrations did not differ. In contrast, fever particles were found in the protected coastal lagoon of Los Pantanos de Villa in Peru which is exposed to similar sources of contamination of the lagoonal system of Magdalena (Losno-Prado & Iannacone, 2023). Moreover, when comparing two remote and less populated areas such as Galápagos Islands and Cocos Island, MP were less abundant than in areas close to the continent (Jones et al., 2021; Astorga et al., 2022). Furthermore, in the Atlantic coast of South America, similar levels of MP pollution have been found in the Argentinian marine platform (Ronda et al., 2019) and in the continental shelf of Rio de Janeiro state (Baptista et al., 2019b) which are exposed to fishery, tourism, untreated sewage and industrial and harbor dredging, while, the lower amounts in Bahía de Vitória in Brazil were related to different hydrodynamic conditions and sediment characteristics as well as less human activities (Baptista et al., 2019a). In contrast, in the Brazilian Santos São Vicente estuary, the abundance of MP was higher in places where silting sites and low energy areas are densely populated and a precarious garbage collection system is dominant, factors that were considered as key for a hotspot for MP pollution in sediments (Fernandes et al., 2025).

**Figure 7:**
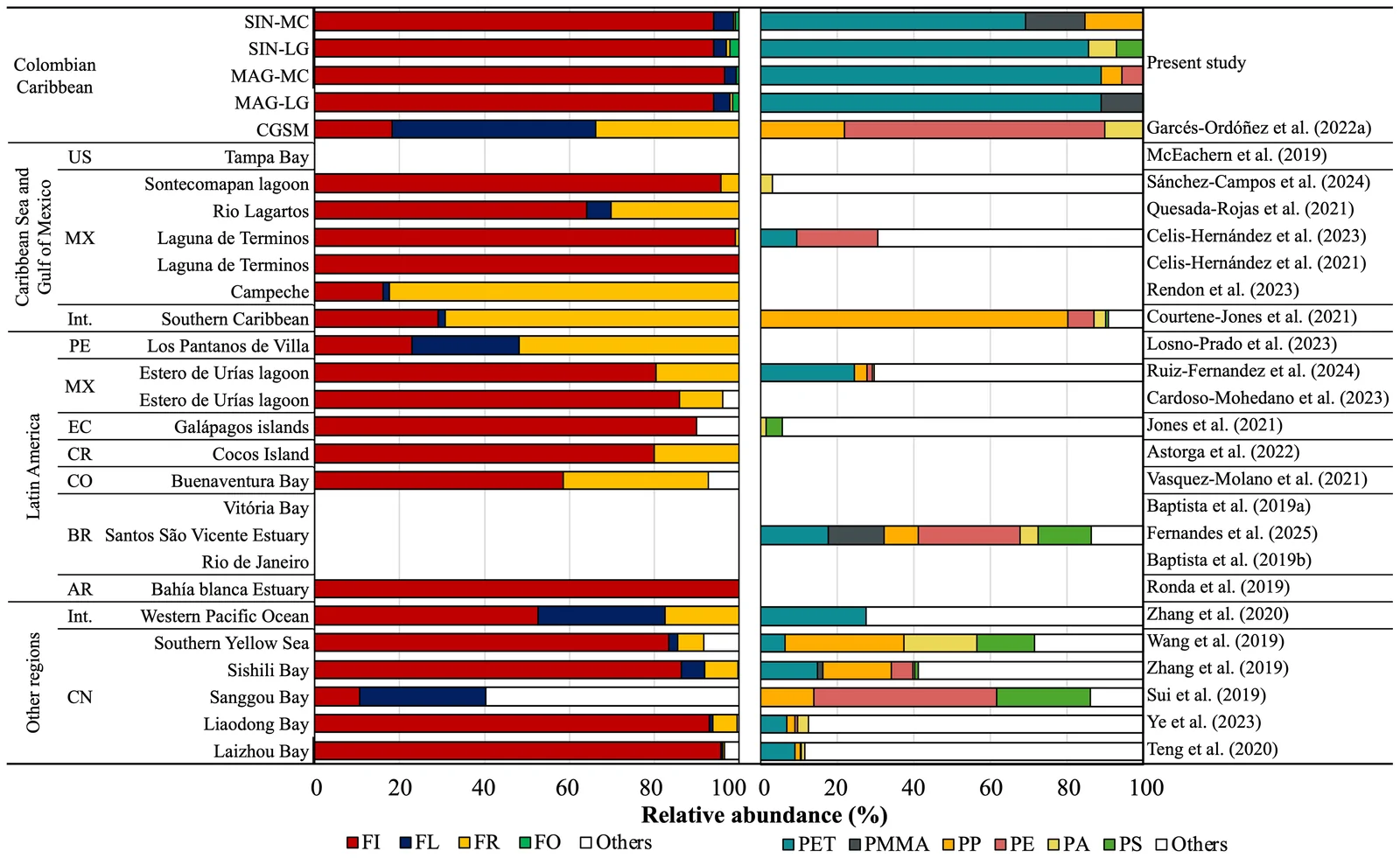
Relative abundance of microplastics in marine sediments reported in different studies compared to this research according to the shapes and polymers. Shapes (FI, Fiber; FL, Film; FO, Foam; FR, Fragment) and polymers (PET, Polyethylene terephthalate; PMMA, Polymethyl methacrylate; PP, Polypropylene; PE, Polyethylene; PA, Polyamide; PS, Polystyrene). Others: refers to different shapes or polymers that were registered in other studies but in this one. SIN, Sinú; MAG, Magdalena; MC, Marine-coastal environment; LG, Lagoonal environment; CGS, Ciénaga Grande de Santa Marta; MX, Mexico; PE, Peru; EC, Ecuador; CR, Costa Rica; CO, Colombia; BR, Brasil; AR, Argentina and CN, China. The data used for this figure is fully described in Table S6.

The prevalence of secondary microplastics found in all the environments, and specifically of fiber, follows the tendency exhibited in the CGSM (Garcés-Ordóñez et al., 2022b) and in the Colombian Pacific, where they were found to account for between 63.7% and 56. 03% of the total in 2015 and 2019, respectively (Vásquez-Molano, Molina & Duque, 2021). This also coincides with the findings of this research and the ones conducted by several studies as shown in Fig. 7 and Table S6, such as the one of Orona-Návar et al. (2022) on Latin America and the Caribbean sediments whose study concluded that this was the most abundant shape of MP (49%), followed by fragments (29%), pellets (17%) and films (5%). The greater presence of fibers in the sediment is strongly influenced by some well-known contaminant sources, such as residual waters associated with port facilities (Claessens et al., 2011; Baptista et al., 2019a), which are channeled through underwater outfall pipes (Baptista et al., 2019b). Concerning this kind of MP, Zobkov & Esiukova (2017) and Ronda et al. (2019) found that concentrations are independent of local environmental parameters and the characteristics of the sediment (granulometry, water content, organic material and depth) but rather they are explained by human activities and hydrodynamic conditions which act as factors that may lead to the movement of plastics in the water column and therefore to the dispersal and distribution of MP (Quesada-Rojas, Enriquez & Valle-Levinson, 2021; Sánchez-Campos et al., 2024; Wu et al., 2025). Nevertheless, according to other study carried out along the Caribbean, the fragments were the dominant shape in the sediments around Antigua (52–93%) and Panamá (73%), however there were more fibers (60%) in Aruba (Courtene-Jones et al., 2021) as in all the locations of this research. However, in the review of Garcés-Ordóñez et al. (2022a), concluded that the most common shapes of MP in sediments of coastal lagoons are both fragments and fibers, which were respectively related to any kind of hard plastic item that undergoes environmental fragmentation, and to wastewater discharges and fishing gear (nets and ropes). In the same study, films were the third most common shape in sediments after fibers and fragments, tendency that was similar in the present study, but in fragment abundance which was lower than the one of films.

### Comparisons with other regions

Most of MP studies are focused on Europe, accounting for around 30% of the global scientific production, however, the greatest amount of research is concerned about China reaching up to 21% of the total (Orona-Návar et al., 2022). This is because of the amount of plastic pollution that lies there, for example, the Yellow Sea, located between China and the Korean Peninsula, has been described as a highly and continuously human-impacted ecosystem that faces industrial activity, massive tourism, artisanal and industrial fishing and aquiculture (Wang et al., 2019; Li & Sun, 2020). Moreover, this region limits with the Bohai Sea to the north and the East China Sea to the south, where the Yangtze River mouth is located, which according to Lebreton et al. (2017) is one of the most polluted with plastic particles around the world. Consequently, the western Pacific tends to reach the highest concentrations of MP compared to other regions of the world. In fact, the amount of these particles increases, as the studies are closer to China (Fig. 6, Table S6).

The dominance of fibers is similar to the levels reported in global studies, in which they have been found to represent between 50% and 80% of all isolated forms (Graca et al., 2017; Wang et al., 2019; Teng et al., 2020; Zhang et al., 2020; Ye et al., 2023). According to Zhang et al. (2019), the greatest abundance of fibers in areas close to ports, river mouths and residual water outfall pipes rather than in the open sea, suggests that they derive principally from terrestrial sources. Additionally, Teng et al. (2020) suggest that the higher proportion of fibers (and films) in the sediment may be explained by the fact that these particles have a larger surface area to volume ratio, leading to higher rates of aggregation/bioincrustation, which cause them to sink more quickly than larger fragments (Ryan, 2015).

The bathymetric range of the locations did not exceed 30 m, and silts dominated. However, no correlation was found between the concentration of MP and grain size. Ramírez-Álvarez et al. (2019) suggested that bathymetry could explain the distribution of MP, with greater concentrations found in sediments lying deeper than 100 m, as these sediments are sandy silts. However, no differences were found between the size of sediment grains and the deposition of MP, a finding that agrees with those of this study.

### Characterization of polymers

Because of their large surface areas and hydrophobic nature, MP are capable of adsorbing a range of environmental contaminants (Wang et al., 2018). Furthermore, their adsorption capacity increases as they age (Ding et al., 2020). Different kinds of polymers have been reported among the MP found in marine sediments (Fig. 7). Particularly, in coastal lagoons around the globe polyethylene, polyester and polypropylene seem to be the predominant polymers (Garcés-Ordóñez et al., 2022a), however, in the Ciénaga Grande de Santa Marta, in the Colombian Caribbean, the most abundant were found to be polypropylene, polyethylene and high- density polyethylene (Garcés-Ordóñez et al., 2022b). In the southern Caribbean, polypropylene is the most abundant (Courtene-Jones et al., 2021), while in other regions of the world, alkyd resin predominates in the Bahía de Todos Santos (Mexico) (Ramírez-Álvarez et al., 2019), polyester (14%) in the Galapagos Islands (Jones et al., 2021), polypropylene (31%) and polyester (24%) in the southern Yellow Sea (Wang et al., 2019), rayon (58% and 87.5%) in the northern Yellow Sea and in Bohai Sea, respectively (Zhang et al., 2019; Ye et al., 2023), and the ethylene-propylene copolymer (40%) and terephthalate of polyethylene (27%) in the western Pacific (Zhang et al., 2020) and in Estero de Urias coastal lagoon (Ruiz-Fernández et al., 2024) where it appeared in three sediment records since the 1970s, which was linked to the widespread use of PET bottles after some multinationals began bottling carbonated beverages. Additionally, due its density (1.37–1.45 g/cm^3^), it tends to sink and accumulate faster than less dense polymers like PP and PE (Isaac & Kandasubramanian, 2021; Zhang et al., 2020), which is consistent with the results of this research.

PET is considered to be a polymer without negative effects on human health, as it used extensively in food and drink packaging as well as electronical and automobile parts, sporting equipment and textiles (Sinha, Patel & Patel, 2010; Webb et al., 2013; Singh et al., 2018; Piccardo et al., 2020). However, recent studies of fresh water species have demonstrated the bioavailability and toxicity of this polymer. For example, in the marine environment, PET—alongside polyvinylchloride and polylactic acid—has been associated with a 10% reduction feeding rate of the polychaeta *Arenicola marina*, affecting the role it plays in the bioturbation and oxygenation of sediments (Green et al., 2016). The authors of this study also found that the three polymers resulted in a significant reduction in microalgal biomass in sediments, potentially reducing the primary productivity of marine ecosystems. Similarly, high concentrations of PET affect ecosystem functioning by altering net flows of nitrogen compounds and the organic content of sediments, modifying the nutrient cycle (You, Trush & Hope, 2020). These alterations might be magnified in the near future, affecting different environmental conditions, among which reductions in pH might be of particular importance, as they could modify the chemical balance of MP, increasing or reducing the lixiviation rate of chemical substances found on their surfaces, producing unpredictable changes in ecotoxicological parameters (Piccardo et al., 2020).

## Conclusions

Microplastics were present in the sediments collected in all samples. Pieces measuring 0.3–1.4 mm were the most abundant as were secondary MP, the most abundant being fibers, which have been associated with the degradation of fishing nets, the breakdown of textile products and discharges of residual waters. Moreover, as PET was the most common polymer, MP pollution in marine-coastal and lagoonal environments might mirror precarious wastewater and sewage systems as well as mismanaged maritime activities due the relation of this polymer to urban runoff and consumer goods and fishing materials. Despite significant differences were found between seasons, this factor had different effects in the environments of Magdalena and Sinú, showing that either proximity to pollution sources, hydrodynamics and human activities can modulate how seasonal changes influences MP pollution in sediments. Particle concentration was greater in the Magdalena environments, which is affected permanently by the river after which it is named and its riverine settlements (lagoonal), and important tourist centers because of their beaches such as El Rodadero and Santa Marta (marine-coastal), with the result that plastic contaminants affect the marine sediments in these areas.

##  Supplemental Information

10.7717/peerj.21127/supp-1 Supplemental Information 1 Raw data

10.7717/peerj.21127/supp-2 Supplemental Information 2 Characterization and quantification of microplastics in marine sediments of the Colombian Caribbean

## Additional Information and Declarations

### Data Availability

The following information was supplied regarding data availability: The raw data is available at Zenodo: Garzón -Rodríguez, L., Tigreros, P., Ochoa-Mogollón, J. D., Herrera Villarraga, G., Sarmiento Sanchez, M. C., Polania Zenner, P. I., Rozo-Torres, G., Sanjuan-Muñoz, A., & Franco-Herrera, A. (2026). Microplastics of marine sediments of the Colombian Caribbean [Data set]. Zenodo. https://doi.org/10.5281/zenodo.18209096. The raw measurements are available in the Supplemental Files.
