# Supplementary material for: Characterization and quantification of microplastics in marine sediments of the Colombian Caribbean
Source: PeerJ. 2026 Sep 2;14:e21127. doi: 10.7717/peerj.21127 (PMC13546627; doi:10.7717/peerj.21127)
Supplement: Supplementary file 1 [file peerj-14-21127-s002.docx]

**Characterization and quantification of microplastics in marine sediments of the Colombian Caribbean**

Luis Felipe Garzón-Rodríguez^1^, Paulo Tigreros-Benavides^1^, Jesús Ochoa-Mogollón^1^, Gysseth Herrera-Villarraga^1^, Camila Sarmiento-Sánchez^1^, Paula Polanía-Zenner^1^, Gladys Rozo-Torres^1^, Adolfo Sanjuan-Muñoz^1^, Andrés Franco-Herrera^1^

^1^ Área de Ciencias Biológicas y Ambientales, Facultad de Ciencias Naturales e Ingeniería, Universidad de Bogotá Jorge Tadeo Lozano, Santa Marta, Colombia

Corresponding Author:

Luis Garzón-Rodríguez^1^

Carrera 2 # 11-68, Edificio Mundo Marino. El Rodadero, Santa Marta, Colombia

Email address: luisf.garzonr@utadeo.edu.co

Supplementary material

**Table S 1.** Coordinates of the stations sampled and locations in the lagoonal and marine-coastal environments of Magdalena and Sinú sectors (WGS84 EPSG: 4326).

| Sector | Environment | Location | Station code | Longitude  (W) | Latitude  (N) | Description |
| --- | --- | --- | --- | --- | --- | --- |
| Magdalena | Marine-coastal | Atlántico | ATL-1 | 75° 07' 53.5'' | 10° 54' 00.8'' | International port |
|  |  |  | ATL-2 | 75° 04' 22.2'' | 10° 55' 22.2'' |  |
|  |  |  | ATL-3 | 75° 02' 51.2'' | 10° 55' 27.3'' |  |
|  |  | El Rodadero | ROD-1 | 74° 15' 17.1'' | 11° 10' 45.1'' | Coal ports, touristic beaches |
|  |  |  | ROD-2 | 74° 14' 23.0'' | 11° 12' 08.4'' |  |
|  |  |  | ROD-3 | 74° 14' 24.5'' | 11° 13' 26.1'' |  |
|  |  | Salamanca | SAL-1 | 74° 39' 31.3'' | 11° 04' 50.6'' | Artisanal fishing |
|  |  |  | SAL-2 | 74° 36' 11.9'' | 11° 02' 50.6'' |  |
|  |  |  | SAL-3 | 74° 30' 56.4'' | 11° 03' 58.7'' |  |
|  |  | Tasajera | TAS-1 | 74° 25' 32.3'' | 11° 00' 18.8'' | Artisanal fishing |
|  |  |  | TAS-2 | 74° 22' 30.4'' | 11° 01' 27.7'' |  |
|  |  |  | TAS-3 | 74° 18' 56.2'' | 11° 04' 21.6'' |  |
|  | Lagoonal | Ciénaga Grande de Santa Marta | CGS-1 | 74° 29' 33.8'' | 10° 57' 04.9'' | Mangrove forest, artisanal fishing |
|  |  |  | CGS-2 | 74° 21' 25.8'' | 10° 58' 07.7'' |  |
|  |  |  | CGS-3 | 74° 18' 03.8'' | 10° 58' 22.1'' |  |
| Sinú | Marine-coastal | Isla Fuerte | ISF-1 | 76° 04' 56.8'' | 09° 25' 29.6'' | Touristic beaches |
|  |  |  | ISF-2 | 76° 03' 01.4'' | 09° 26' 34.7'' |  |
|  |  |  | ISF-3 | 76° 00' 47.7'' | 09° 27' 17.6'' |  |
|  |  | Mestizos | MES-1 | 75° 52' 00.8'' | 09° 28' 33.6'' | Fuel port terminal, touristic beaches |
|  |  |  | MES-2 | 75° 49' 55.3'' | 09° 28' 48.2'' |  |
|  |  |  | MES-3 | 75° 47' 27.3'' | 09° 29' 17.0'' |  |
|  |  | Tinajones | TIN-1 | 75° 58' 51.5'' | 09° 26' 48.7'' | Artisanal fishing |
|  |  |  | TIN-2 | 75° 56' 41.8'' | 09° 27' 19.3'' |  |
|  |  |  | TIN-3 | 75° 54' 13.3'' | 09° 27' 29.0'' |  |
|  | Lagoonal | La Caimanera | CAI-1 | 75° 37' 31.1'' | 09° 25' 00.4'' | Mangrove forest, ecotourism |
|  |  |  | CAI-2 | 75° 37' 23.7'' | 09° 25' 29.3'' |  |
|  |  |  | CAI-3 | 75° 37' 41.2'' | 09° 25' 55.5'' |  |
|  |  | Cispatá | CIS-1 | 75° 48. 50.6'' | 09° 24' 04.0'' | Artisanal fishing |
|  |  |  | CIS-2 | 75° 48' 26.6'' | 09° 24' 08.0'' |  |
|  |  |  | CIS-3 | 75° 47' 43.0'' | 09° 23' 38.7'' |  |

**Table S 2.** Results of the marginal tests of the distance-based linear models (DistLM) of sediment grain size as prediction variables for the concentration of microplastics of 0.3-1.4 mm and 1.4-5.0 mm in the lagoonal and marine-coastal environment.

| **Size** | **Environment** | **Predictive variable** | **Square sum** | **Pseudo-*F*** | ***p*-value** | **Percentage explained** |
| --- | --- | --- | --- | --- | --- | --- |
| 0.3-1.4 mm | Lagoonal | < 63 µm | 1530.9 | 1.70 | 0.1956 | 9.58% |
|  |  | 63 µm | 749.6 | 0.79 | 0.4340 | 4.69% |
|  |  | 90 µm | 1097.1 | 1.18 | 0.3104 | 6.87% |
|  |  | 125 µm | 2014.4 | 2.31 | 0.1036 | 12.61% |
|  |  | 180 µm | 1414.9 | 1.55 | 0.1971 | 8.85% |
|  |  | 250 µm | 454.8 | 0.47 | 0.5302 | 2.85% |
|  |  | 500 µm | 1444.0 | 1.59 | 0.2011 | 9.04% |
|  |  | 1000 µm | 254.4 | 0.26 | 0.8173 | 1.59% |
|  | Marine-coastal | < 63 µm | 465.4 | 0.58 | 0.5176 | 1.44% |
|  |  | 63 µm | 2067.6 | 2.73 | 0.0872 | 6.39% |
|  |  | 90 µm | 889.6 | 1.13 | 0.2969 | 2.75% |
|  |  | 125 µm | 822.3 | 1.04 | 0.3325 | 2.54% |
|  |  | 180 µm | 116.8 | 0.15 | 0.8609 | 0.36% |
|  |  | 250 µm | 124.5 | 0.15 | 0.8470 | 0.38% |
|  |  | 500 µm | 1324.7 | 1.71 | 0.1904 | 4.10% |
|  |  | 1000 µm | 179.2 | 0.22 | 0.7916 | 0.55% |
| 1.4-5.0 mm | Lagoonal | < 63 µm | 184.9 | 0.16 | 0.8430 | 1.00% |
|  |  | 63 µm | 559.2 | 0.50 | 0.5871 | 3.03% |
|  |  | 90 µm | 201.5 | 0.18 | 0.8287 | 1.09% |
|  |  | 125 µm | 110.0 | 0.10 | 0.9454 | 0.60% |
|  |  | 180 µm | 101.0 | 0.09 | 0.9373 | 0.55% |
|  |  | 250 µm | 1005.0 | 0.92 | 0.4168 | 5.44% |
|  |  | 500 µm | 1229.6 | 1.14 | 0.3103 | 6.66% |
|  |  | 1000 µm | 1803.6 | 1.73 | 0.1790 | 9.77% |
|  | Marine-coastal | < 63 µm | 1375.2 | 1.06 | 0.3221 | 2.70% |
|  |  | 63 µm | 3857.9 | 3.12 | 0.0610 | 7.58% |
|  |  | 90 µm | 944.1 | 0.72 | 0.4568 | 1.86% |
|  |  | 125 µm | 1144.0 | 0.87 | 0.3949 | 2.25% |
|  |  | 180 µm | 1080.1 | 0.82 | 0.4256 | 2.12% |
|  |  | 250 µm | 133.9 | 0.10 | 0.9200 | 0.26% |
|  |  | 500 µm | 403.6 | 0.30 | 0.7362 | 0.79% |
|  |  | 1000 µm | 2656.5 | 2.09 | 0.1324 | 5.22% |

**Table S 3.** Results of the sequential tests of the distance-based linear models (DistLM) of sediment grain size as prediction variables for the concentration of microplastics of 0.3-1.4 mm and 1.4-5.0 mm in the lagoonal and marine-coastal environment.

| **Size** | **Environment** | **Predictive variable** | **R^2^ adjusted** | **Square sum** | **Pseudo-*F*** | ***p*-value** | **Percentage** | **Accumulated percentage** | **Residual d.f.** |
| --- | --- | --- | --- | --- | --- | --- | --- | --- | --- |
| 0.3-1.4 mm | Lagoonal | < 63 µm | 0.04 | 1530.9 | 1.70 | 0.1888 | 9.58% | 9.58% | 16 |
|  |  | 63 µm | 0.01 | 526.3 | 0.57 | 0.5439 | 3.29% | 12.87% | 15 |
|  |  | 90 µm | -0.01 | 627.7 | 0.66 | 0.4773 | 3.93% | 16.80% | 14 |
|  |  | 125 µm | 0.13 | 2689.7 | 3.30 | 0.0655 | 16.83% | 33.64% | 13 |
|  |  | 180 µm | 0.12 | 636.9 | 0.77 | 0.4518 | 3.99% | 37.62% | 12 |
|  |  | 250 µm | 0.05 | 131.6 | 0.15 | 0.8597 | 0.82% | 38.44% | 11 |
|  |  | 500 µm | 0.09 | 1279.4 | 1.50 | 0.2354 | 8.01% | 46.45% | 10 |
|  |  | 1000 µm | 0.09 | -3.0×10^-12^ | N/A | N/A | 0.00% | 46.45% | 10 |
|  | Marine-coastal | < 63 µm | -0.01 | 465.4 | 0.58 | 0.5129 | 1.44% | 1.44% | 40 |
|  |  | 63 µm | 0.03 | 1938.7 | 2.53 | 0.0949 | 5.99% | 7.43% | 39 |
|  |  | 90 µm | 0.00 | 60.6 | 0.08 | 0.9354 | 0.19% | 7.62% | 38 |
|  |  | 125 µm | 0.03 | 1487.1 | 1.94 | 0.1564 | 4.60% | 12.22% | 37 |
|  |  | 180 µm | 0.01 | 413.0 | 0.53 | 0.5469 | 1.28% | 13.50% | 36 |
|  |  | 250 µm | 0.00 | 380.0 | 0.48 | 0.5713 | 1.17% | 14.67% | 35 |
|  |  | 500 µm | 0.03 | 1539.6 | 2.01 | 0.1573 | 4.76% | 19.43% | 34 |
|  |  | 1000 µm | 0.03 | 3.9×10^-12^ | N/A | N/A | 0.00% | 19.43% | 34 |
| 1.4-5.0 mm | Lagoonal | < 63 µm | -0.05 | 184.9 | 0.16 | 0.8404 | 1.00% | 1.00% | 16 |
|  |  | 63 µm | -0.09 | 505.0 | 0.43 | 0.6328 | 2.73% | 3.74% | 15 |
|  |  | 90 µm | -0.17 | 37.4 | 0.03 | 0.9829 | 0.20% | 3.94% | 14 |
|  |  | 125 µm | -0.25 | 131.7 | 0.10 | 0.9095 | 0.71% | 4.65% | 13 |
|  |  | 180 µm | -0.35 | 8.8 | 0.01 | 0.9986 | 0.05% | 4.70% | 12 |
|  |  | 250 µm | -0.28 | 2350.3 | 1.70 | 0.2099 | 12.73% | 17.43% | 11 |
|  |  | 500 µm | 0.06 | 5057.3 | 4.96 | 0.0302^*^ | 27.39% | 44.81% | 10 |
|  |  | 1000 µm | 0.06 | -6.2×10^-12^ | N/A | N/A | 0.00% | 44.81% | 10 |
|  | Marine-coastal | < 63 µm | 0.00 | 1375.2 | 1.06 | 0.3231 | 2.70% | 2.70% | 38 |
|  |  | 63 µm | 0.04 | 3242.7 | 2.59 | 0.0888 | 6.37% | 9.08% | 37 |
|  |  | 90 µm | 0.04 | 968.8 | 0.77 | 0.4311 | 1.90% | 10.98% | 36 |
|  |  | 125 µm | 0.05 | 1727.1 | 1.39 | 0.2387 | 3.39% | 14.38% | 35 |
|  |  | 180 µm | 0.03 | 684.3 | 0.54 | 0.5360 | 1.35% | 15.72% | 34 |
|  |  | 250 µm | 0.07 | 2651.4 | 2.18 | 0.1283 | 5.21% | 20.93% | 33 |
|  |  | 500 µm | 0.05 | 652.6 | 0.53 | 0.5661 | 1.28% | 22.22% | 32 |
|  |  | 1000 µm | 0.05 | 1.7×10^-11^ | N/A | N/A | 0.00% | 22.22% | 32 |

**Table S 4.** PERMANOVA test for concentration of microplastics by size (Si) season (Se) and environment (En), based on the Bray-Curtis similarity matrix. *: significant differences, d.f.: degrees of freedom. MAG-MC: Magdalena marine-coastal, MAG-LG: Magdalena lagoonal, SIN-MC: Sinú marine-coastal, SIN-LG: Sinú lagoonal.

| **Main test** | | | | | | | | |
| --- | --- | --- | --- | --- | --- | --- | --- | --- |
| **PERMANOVA** | | | | | | **Permdisp** | | |
| **Source** | **d.f.** | **Mean Square** | **Pseudo-*F*** | ***p-*value** | **Unique permutations** | **d.f.** | **Pseudo-*F*** | ***p-*value** |
| Si | 1 | 95272 | 84.26 | 0.0001^*^ | 9946 | 1; 116 | 9.13 | 0.0034^*^ |
| Se | 1 | 4454 | 3.94 | 0.0058 | 9947 | 1; 116 | 3.54 | 0.0569 |
| En | 3 | 1861 | 1.65 | 0.0741 | 9924 | 3; 114 | 1.19 | 0.3009 |
| Si×Se | 1 | 1632 | 1.44 | 0.2180 | 9942 | 3; 114 | 4.06 | 0.0087^*^ |
| Si×En | 3 | 2075 | 1.84 | 0.0460^*^ | 9937 | 7; 110 | 2.09 | 0.0435^*^ |
| Se×En | 3 | 1155 | 1.02 | 0.4183 | 9925 | 7; 110 | 0.99 | 0.3808 |
| Si×Se×En | 3 | 1960 | 1.73 | 0.0553 | 9923 | 15;102 | 0.97 | 0.4577 |
| Res | 102 | 1131 |  |  |  |  |  |  |
| Total | 117 |  |  |  |  |  |  |  |
| **Pairwise test Si×En** | | | | | | | | |
| **PERMANOVA** | | | | | | | **Permdisp** | |
| **Samples** | | | | **Pseudo *t*** | ***p-*value** | **Unique permutations** | **Pseudo-*F*** | ***p-*value** |
| 1.4-5.0 mm MAG-MC × 0.3-1.4 mm MAG-MC | | | | 5.62 | 0.0001 | 9939 | 0.90 | 0.3795 |
| 1.4-5.0 mm MAG-LG × 0.3-1.4 mm MAG-LG | | | | 3.36 | 0.0001 | 7054 | 0.19 | 0.8536 |
| 1.4-5.0 mm SIN-MC × 0.3-1.4 mm SIN-MC | | | | 6.07 | 0.0001 | 9942 | 2.40 | 0.0201 |
| 1.4-5.0 mm SIN-LG × 0.3-1.4 mm SIN-LG | | | | 5.85 | 0.0001 | 9948 | 2.13 | 0.0545 |
| 1.4-5.0 mm MAG-MC × 1.4-5.0 mm MAG-LG | | | | 0.82 | 0.5170 | 9952 | 1.91 | 0.0531 |
| 1.4-5.0 mm MAG-MC × 1.4-5.0 mm SIN-MC | | | | 1.67 | 0.0443^*^ | 9947 | 0.42 | 0.6741 |
| 1.4-5.0 mm MAG-MC × 1.4-5.0 mm SIN-LG | | | | 1.76 | 0.0478^*^ | 9957 | 0.43 | 0.6540 |
| 1.4-5.0 mm MAG-LG × 1.4-5.0 mm SIN-MC | | | | 1.17 | 0.2371 | 9937 | 1.53 | 0.1266 |
| 1.4-5.0 mm MAG-LG × 1.4-5.0 mm SIN-LG | | | | 1.54 | 0.0920 | 9948 | 1.80 | 0.0750 |
| 1.4-5.0 mm SIN-MC × 1.4-5.0 mm SIN-LG | | | | 0.51 | 0.9066 | 9936 | 0.03 | 0.9755 |
| 0.3-1.4 mm MAG-MC × 0.3-1.4 mm MAG-LG | | | | 0.37 | 0.9135 | 9954 | 0.35 | 0.7222 |
| 0.3-1.4 mm MAG-MC × 0.3-1.4 mm SIN-MC | | | | 1.44 | 0.1195 | 9940 | 2.02 | 0.0554 |
| 0.3-1.4 mm MAG-MC × 0.3-1.4 mm SIN-LG | | | | 1.07 | 0.3020 | 9944 | 1.37 | 0.1917 |
| 0.3-1.4 mm MAG-LG × 0.3-1.4 mm SIN-MC | | | | 1.04 | 0.3372 | 9933 | 1.76 | 0.0977 |
| 0.3-1.4 mm MAG-LG × 0.3-1.4 mm SIN-LG | | | | 1.19 | 0.2416 | 9953 | 1.19 | 0.2709 |
| 0.3-1.4 mm SIN-MC × 0.3-1.4 mm SIN-LG | | | | 2.09 | 0.0301 | 9956 | 0.26 | 0.7942 |

**Table S 5.** Average ± standard deviation (minimum-maximum) of the concentration of microplastics (MP/kg) in the localities of all the environments (Environ.) during the two climatic periods, by shape. - : no MP recorded.

| **Size**  Environ. | **Locality** | **Rainy season** | | | | | **Dry season** | | | | |
| --- | --- | --- | --- | --- | --- | --- | --- | --- | --- | --- | --- |
|  |  | **FO** | **FI** | **FR** | **FL** | **MP** | **FO** | **FI** | **FR** | **FL** | **MP** |
| 0.3-1.4 mm  MAG-MC | ATL | 1.1±1.9 (0.0-3.3) | 127.8±33.4 (93.3-160.0) | - | 2.2±1.9 (0.0-3.3) | 131.1±36.7 (93.3-166.7) | - | 143.3±101.7 (43.3-246.7) | - | 2.2±1.9 (0.0-3.3) | 145.6±101.8 (46.7-250.0) |
|  | ROD | 3.3±3.3 (0.0-6.7) | 210.0±122.5 (80.0-323.3) | - | 3.3±3.3 (0.0-6.7) | 216.7±127.2 (83.3-336.7) | 3.3±0.0 (3.3-3.3) | 303.3±138.6 (153.3-426.7) | 1.1±1.9 (0.0-3.3) | 11.1±5.1 (6.7-16.7) | 318.9±136.2 (173.3-443.3) |
|  | SAL | - | 52.2±28.3 (23.3-80.0) | - | 1.1±1.9 (0.0-3.3) | 53.3±28.5 (23.3-80.0) | - | 185.6±49.9 (130.0-226.7) | - | 2.2±3.8 (0.0-6.7) | 187.8±46.2 (136.7-226.7) |
|  | TAS | 1.1±1.9 (0.0-3.3) | 62.2±36.0 (36.7-103.3) | - | - | 63.3±37.9 (36.7-106.7) | - | 123.3±80.9 (33.3-190.0) | - | 1.1±1.9 (0.0-3.3) | 124.4±79.0 (36.7-190.0) |
| 0.3-1.4 mm  MAG-LG | CGS | 1.1±1.9 (0.0-3.3) | 67.8±54.0 (33.3-130.0) | - | 2.2±3.8 (0.0-6.7) | 71.1±59.8 (33.3-140.0) | 3.3±3.3 (0.0-6.7) | 245.6±201.1 (110.0-476.7) | - | 10.0±11.5 (3.3-23.3) | 258.9±209.6 (120.0-500.0) |
| 0.3-1.4 mm  SIN-MC | ISF | 1.1±1.9 (0.0-3.3) | 85.6±17.1 (66.7-100.0) | 1.1±1.9 (0.0-3.3) | 1.1±1.9 (0.0-3.3) | 88.9±13.9 (73.3-100.0) | - | 73.3±6.7 (66.7-80.0) | - | 1.1±1.9 (0.0-3.3) | 74.4±6.9 (66.7-80.0) |
|  | MES | 1.1±1.9 (0.0-3.3) | 131.1±75.7 (50.0-200.0) | - | - | 132.2±76.0 (50.0-200.0) | 3.3±5.8 (0.0-10.0) | 91.1±29.1 (66.7-123.3) | - | 13.3±11.5 (0.0-20.0) | 107.8±31.5 (83.3-143.3) |
|  | TIN | - | 130.0±85.7 (63.3-226.7) | 1.1±1.9 (0.0-3.3) | 14.4±1.9 (13.3-16.7) | 145.6±86.3 (80.0-243.3) | - | 148.9±57.4 (83.3-190.0) | - | 4.4±5.1 (0.0-10.0) | 153.3±57.8 (86.7-190.0) |
| 0.3-1.4 mm  SIN-LG | CIS | 2.2±3.8 (0.0-6.7) | 83.3±45.1 (40.0-130.0) | 2.2±3.8 (0.0-6.7) | 2.2±3.8 (0.0-6.7) | 90.0±55.7 (40.0-150.0) | - | 153.3±41.8 (110.0-193.3) | - | 3.3±3.3 (0.0-6.7) | 156.7±43.7 (110.0-196.7) |
|  | CAI | 5.6±1.9 (3.3-6.7) | 268.9±71.7 (196.7-340.0) | 3.3±5.8 (0.0-10.0) | 8.9±15.4 (0.0-26.7) | 286.7±76.9 (200.0-346.7) | 2.2±1.9 (0.0-3.3) | 177.8±39.1 (133.3-206.7) | 1.1±1.9 (0.0-3.3) | 6.7±6.7 (0.0-13.3) | 187.8±41.4 (140.0-213.3) |
| 1.4-5.0 mm  MAG-MC | ATL | - | 6.7±3.3 (3.3-10.0) | - | - | 6.7±3.3 (3.3-10.0) | - | 26.7±23.3 (10.0-53.3) | - | - | 26.7±23.3 (10.0-53.3) |
|  | ROD | - | 15.6±11.7 (3.3-26.7) | - | 5.6±9.6 (0.0-16.7) | 21.1±20.4 (3.3-43.3) | - | 31.1±21.2 (6.7-43.3) | 1.1±1.9 (0.0-3.3) | 3.3±3.3 (0.0-6.7) | 35.6±19.5 (13.3-50.0) |
|  | SAL | - | 30.0±5.8 (23.3-33.3) | - | 4.4±5.1 (0.0-10.0) | 34.4±1.9 (33.3-36.7) | - | 8.9±5.1 (3.3-13.3) | - | - | 8.9±5.1 (3.3-13.3) |
|  | TAS | - | 3.3±5.8 (0.0-10.0) | - | - | 3.3±5.8 (0.0-10.0) | - | 23.3±12.0 (13.3-36.7) | - | - | 23.3±12.0 (13.3-36.7) |
| 1.4-5.0 mm  MAG-LG | CGS | - | 14.4±7.7 (10.0-23.3) | 2.2±3.8 (0.0-6.7) | - | 16.7±11.5 (10.0-30.0) | 1.1±1.9 (0.0-3.3) | 13.3±5.8 (10.0-20.0) | - | 2.2±3.8 (0.0-6.7) | 16.7±11.5 (10.0-30.0) |
| 1.4-5.0 mm  SIN-MC | ISF | - | 7.8±5.1 (3.3-13.3) | - | - | 7.8±5.1 (3.3-13.3) | - | 4.4±1.9 (3.3-6.7) | - | - | 4.4±1.9 (3.3-6.7) |
|  | MES | 1.1±1.9 (0.0-3.3) | 12.2±8.4 (3.3-20.0) | - | - | 13.3±10.0 (3.3-23.3) | - | 7.8±8.4 (0.0-16.7) | - | 1.1±1.9 (0.0-3.3) | 8.9±6.9 (3.3-16.7) |
|  | TIN | - | 17.8±12.6 (3.3-26.7) | - | 1.1±1.9 (0.0-3.3) | 18.9±13.9 (3.3-30.0) | - | 4.4±3.8 (0.0-6.7) | 1.1±1.9 (0.0-3.3) | - | 5.6±1.9 (3.3-6.7) |
| 1.4-5.0 mm  SIN-LG | CIS | - | 11.1±10.7 (3.3-23.3) | - | - | 11.1±10.7 (3.3-23.3) | - | 5.6±1.9 (3.3-6.7) | - | - | 5.6±1.9 (3.3-6.7) |
|  | CAI | 5.6±5.1 (0.0-10.0) | 24.4±13.5 (10.0-36.7) | - | - | 30.0±15.3 (16.7-46.7) | 1.1±1.9 (0.0-3.3) | 3.3±0.0 (3.3-3.3) | - | 2.2±1.9 (0.0-3.3) | 6.7±3.3 (3.3-10.0) |
| Total  MAG-MC | ATL | 1.1±1.9 (0.0-3.3) | 134.4±36.7 (96.7-170.0) | - | 2.2±1.9 (0.0-3.3) | 137.8±40.0 (96.7-176.7) | - | 170.0±123.9 (53.3-300.0) | - | 2.2±1.9 (0.0-3.3) | 172.2±124.1 (56.7-303.3) |
|  | ROD | 3.3±3.3 (0.0-6.7) | 225.6±117.5 (96.7-326.7) | - | 8.9±10.2 (0.0-20.0) | 237.8±123.9 (100.0-340.0) | 3.3±0.0 (3.3-3.3) | 334.4±158.6 (160.0-470.0) | 2.2±3.8 (0.0-6.7) | 14.4±8.4 (6.7-23.3) | 354.4±155.4 (186.7-493.3) |
|  | SAL | - | 82.2±23.6 (56.7-103.3) | - | 5.6±3.8 (3.3-10.0) | 87.8±26.7 (60.0-113.3) | - | 194.4±45.4 (143.3-230.0) | - | 2.2±3.8 (0.0-6.7) | 196.7±41.6 (150.0-230.0) |
|  | TAS | 1.1±1.9 (0.0-3.3) | 65.6±34.2 (36.7-103.3) | - | - | 66.7±36.1 (36.7-106.7) | - | 146.7±91.7 (46.7-226.7) | - | 1.1±1.9 (0.0-3.3) | 147.8±89.8 (50.0-226.7) |
| Total  MAG-LG | CGS | 1.1±1.9 (0.0-3.3) | 82.2±51.0 (43.3-140.0) | 2.2±3.8 (0.0-6.7) | 2.2±3.8 (0.0-6.7) | 87.8±55.5 (43.3-150.0) | 4.4±1.9 (3.3-6.7) | 258.9±206.9 (120.0-496.7) | - | 12.2±15.4 (3.3-30.0) | 275.6±221.1 (130.0-530.0) |
| Total  SIN-MC | ISF | 1.1±1.9 (0.0-3.3) | 93.3±20.3 (70.0-106.7) | 1.1±1.9 (0.0-3.3) | 1.1±1.9 (0.0-3.3) | 96.7±17.3 (76.7-106.7) | - | 77.8±6.9 (70.0-83.3) | - | 1.1±1.9 (0.0-3.3) | 78.9±7.7 (70.0-83.3) |
|  | MES | 2.2±3.8 (0.0-6.7) | 143.3±81.9 (53.3-213.3) | - | - | 145.6±82.8 (53.3-213.3) | 3.3±5.8 (0.0-10.0) | 98.9±36.0 (73.3-140.0) | - | 14.4±9.6 (3.3-20.0) | 116.7±38.4 (86.7-160.0) |
|  | TIN | - | 147.8±95.7 (66.7-253.3) | 1.1±1.9 (0.0-3.3) | 15.6±3.8 (13.3-20.0) | 164.4±98.0 (83.3-273.3) | - | 153.3±55.1 (90.0-190.0) | 1.1±1.9 (0.0-3.3) | 4.4±5.1 (0.0-10.0) | 158.9±56.8 (93.3-193.3) |
| Total  SIN-LG | CIS | 2.2±3.8 (0.0-6.7) | 94.4±54.2 (46.7-153.3) | 2.2±3.8 (0.0-6.7) | 2.2±3.8 (0.0-6.7) | 101.1±65.2 (46.7-173.3) | - | 158.9±43.5 (113.3-200.0) | - | 3.3±3.3 (0.0-6.7) | 162.2±45.5 (113.3-203.3) |
|  | CAI | 11.1±5.1 (6.7-16.7) | 293.3±85.0 (206.7-376.7) | 3.3±5.8 (0.0-10.0) | 8.9±15.4 (0.0-26.7) | 316.7±90.6 (216.7-393.3) | 3.3±3.3 (0.0-6.7) | 181.1±39.1 (136.7-210.0) | 1.1±1.9 (0.0-3.3) | 8.9±6.9 (3.3-16.7) | 194.4±41.4 (146.7-220.0) |

**Figure S 1.** Infrared spectrums identified in the MP samples. a) polyethylene terephthalate (PET), b) polypropylene (PP), c) polyethylene terephthalate (PMMA), d) polyethylene (PE), e) polyamide (PA-6) and f) polystyrene (PS).

**Table S 6.** Minimum-maximum or mean±standard deviation of concentrations of microplastics in marine sediments reported in different studies and the relative abundance according to the shapes (FI: Fiber, FL: Film, FO: Foam, FR: Fragment, GR: Granule, PE: Pellet, SP: Spherule) and polymers (ABS: Acrylonitrile butadiene styrene, AC: Acrylic, AR: Alkyl resin, CE: Cellophane, Cell: Cellulose, HDPE: High density polyethylene, PA: Polyamide, PAA: Polyacrylic, PAM: polyacrylamide, PAN: Polyacrylonitrile, PC: Polycarbonate, PE: Polyethylene, PEA: Polyethylene glycol adipate, PES: Polyester, PET: Polyethylene terephthalate, PMMA: Polymethyl-polymethacrylate, PP: Polypropylene, PPA: Polyphthalamide, PS: Polystyrene, PVA: Polyvinyl acetate, PVC: Polyvinyl chloride, RA: Rayon, NI: Not identified). RS: Rainy season, DS: Dry season. N.S.: Not specified.

| **Region** | **Authors** | **Sampling method** | **Depth (m)** | **Particle size (mm)** | **Number of stations** | **Location** | **Concentration** | **Shapes** | **Polymers** |  |
| --- | --- | --- | --- | --- | --- | --- | --- | --- | --- | --- |
|  |  |  |  |  |  |  |  |  |  |  |
| Colombian Caribbean | This study | Van Veen grab sampler | 0.5 - 30.0 | 0.3 - 5.0 | 12 | Magdalena marine-coastal | RS: 36.7 - 340.0 MP/kg | FI: 96.6 %, FL: 2.6 %, FO: 0.6 %, FR: 0.2 %  n = 1261 | PET: 88.9 %, PP: 5.6 %, PE: 5.6 %  n = 18 |  |
|  |  |  |  |  |  |  | DS: 50.0 - 493.3 MP/kg |  |  |  |
|  |  |  |  |  | 3 | Magdalena lagoonal | RS: 43.3 - 150.0 MP/kg | FI: 93.9 %, FL: 4.0 %, FO: 1.5 %, FR: 0.6 %  n = 327 | PET: 88.9 %, PMMA: 11.1 %,   n = 9 |  |
|  |  |  |  |  |  |  | DS: 130.0 - 200.0 MP/kg |  |  |  |
|  |  |  |  |  | 9 | Sinú marine-coastal | RS: 53.3 – 273.3 MP/kg | FI: 93.9 %, FL: 4.8 %, FO: 0.9 %, FR: 0.4 %  n = 685 | PET: 69.2 %, PMMA: 15.4 %, PP: 15.4 %, PE: 5.6 %  n = 13 |  |
|  |  |  |  |  |  |  | DS: 70.0 – 193.0 MP/kg |  |  |  |
|  |  |  |  |  | 6 | Sinú lagoonal | RS: 46.7 – 393.0 MP/kg | FI: 94.0 %, FL: 4.8 %, FO: 2.2 %, FR: 0.9 %  n = 697 | PET: 85.7 %, PA: 7.1 %, PS: 7.1 % n = 14 |  |
|  |  |  |  |  |  |  | DS: 113.3–220.0 MP/kg |  |  |  |
|  | Garcés-Ordóñez et al. (2022b) | Van Veen grab sampler | 0.7 - 2.2 | 0.5 - 5.0 | 33 | Ciénaga Grande de Santa Marta | 0 - 3.1 items/kg | FL: 48.0 %, FR: 33.8 %, FI: 18.2 % n = 77 | PA: 10.0 %, HDPE: 50.0 %, PP: 22.0 %, PE: 18.0 % n = 24 |  |
| Caribbean Sea and Gulf of Mexico | Borges et al. (2019) | Van Veen grab sampler | N.S. | 0.24 - 5.16 | 15 | Coast of Campeche state, Mexico | 0 - 800 items/m^2^ | FR: 21.2 %, FI: 2.2 %, Paint: 76.6 %  n = 5220 | N.S. |  |
|  | McEachern et al. (2019) | Shipek grab sampler | 2.6 - 11.0 | 0.053 - 5.0 | 9 | Tampa bay, United States of America | 30 - 790 particles/kg | N.S. | N.S. |  |
|  | Celis-Hernández et al. (2021) | Hand-held aluminum corer | 1.1 - 1.45 | 2-3 - 6.2 | 6 | Laguna de Terminos, Mexico (Gulf of Mexico) | 4 - 28 items/kg | FI | N.S. |  |
|  | Courtene-Jones et al. (2021) | Van Veen grab sampler | 2.7 - 14.4 | 0.08-14.0 | 6 | Southern Caribbean | 0.03 ± 0.003 to 0.13 ± 0.10 MP/g | FR: 69.2 %, FI: 29.0 %, FL: 1.8 %  n = N.S. | PP: 80.3 %, PE: 6.9 %, PVC: 2.9 %, PA: 2.9 %, PES: 2.1 %, AC: 1.7 %, PAA: 1.7 %, PS: 0.8 %, ABS: 0.6 %  n = N.S. |  |
|  | Quesada-Rojas et al. (2021) | Ponar dredge | 0.4 - 3.5 | 0.3 - 5.0 | 30 | Rio Lagartos coastal lagoon, Mexico (Gulf of Mexico) | 0 - 328.1 MP/kg | FI: 64.1 %, FR: 30.2 %, FL: 5.7 % n = N.S. | N.S. |  |
|  | Celis-Hernández et al. (2023) | Van Veen grab sampler | 3.5 | 0.065 - 5.0 | 9 | Laguna de Terminos, Mexico (Southern Gulf of Mexico) | 22.7 – 513.9 MP/g | FI: 99%, FR: 1% n = N.S. | PE: 21.1%, POPD: 12.6 %, PET: 9.5 %, SBR: 8.4 %, PEO: 7.4 %, PVC: 6.3 %, others: 34.7 n = 98 |  |
|  | Rendón-von et al. (2023) | Van Veen grab sampler | 5 - 105 | 0.25 - 5.0 | 124 | Continental shelf of southern Gulf of Mexico | 1.3 - 95.2 MP/kg | FR: 82.4%, FI: 16.2%, FL: 1.4% n = 1105 | PE, PA, PVDF, CE, Nylon, others n = 1105 |  |
|  | Sánchez-Campos et al. (2024) | Van Veen grab sampler | 1.5 | 0.053 - 4.75 | 10 | Sontecomapan coastal lagoon, Mexico (Gulf of Mexico) | 0- 43.01 MP/kg | FI: 95.6%, FR: 4.4%  n = N.S. | PES: 45%, Acrylic: 33 %, Rayon: 19 %, PA: 3 % n = N.S. |  |
| Latin America | Baptista et al. (2019a) | Van Veen grab sampler | 0.5 - 8.0 | N.S. | 20 | Vitória bay estuary, Brazil | 0 - 126.6 particles/kg | FI: 77.0  %, FR: 23.0 %  n = 247 | N.S. |  |
|  | Baptista et al. (2019b) | Van Veen grab sampler | 20 - 55 | 0.2 - 3.0 | 68 | Continental shelf of Rio de Janeiro state, Brazil | 2 - 460 particles/kg | FI: 47.8 %, FL: 28.6 %, FR: 23.1 %, PE: 0.5 %  n = 2400 | N.S. |  |
|  | Ramírez-Álvarez et al. (2019) | Van Veen grab sampler | 30 - 200 | 0.25 - 5.0 | 12 | Todos Santos bay, Mexico (Pacific Ocean) | 85 - 2494 particles/0.1 m^2^ | FR: 73.8 %, FI: 24.4 %; PE:  0.8 %, Others: 1.0 % n = 5667 | AR, PE-PP, PA, PET, PAM, PVC, CE  n = N.S. |  |
|  | Ronda et al. (2019) | Shipek grab sampler | 6.3 - 14.5 | 0.008 - 5.0 | 7 | Argentinean continental shelf (Atlantic Ocean) | 40 - 360 particles/kg | Only fibers sampling | N.S. |  |
|  | Jones et al. (2021) | Van Veen grab sampler | 3.0 - 9.0 | 0.01 - 5.0 | 17 | Galápagos islands, Ecuador (Pacific Ocean) | 6.7 - 86.7 particles/kg | FI > 90 %  n = N.S. | PAM: 59.7 %, PES: 15.3 %, Cell: 13.9 %, Plasticizer: 2.2 %, PVC: 3.3 %, PA: 1.4 %, PS: 4.2 %  n = 86 |  |
|  | Vásquez-Molano et al. (2021) | Box corer | 1.5 – 2.7 | 0.15-3.2 | 36 | Buenaventura Bay, Colombia (Eastern Pacific Ocean) | 11 - 1354 particles/kg | FI: 58.5 %, FR: 34.2 %, PE: 7.2 %  n = N.S. | N.S. |  |
|  | Astorga et al. (2022) | Van Veen grab sampler | 10 | 0.01-5.0 | 4 | Cocos Island, Costa Rica (Pacific Ocean) | 3.35 ± 4.30 MP/kg | FI: 80%, FR: 20%  n = N.S. | N.S. |  |
|  | Cardoso-Mohedano et al. (2023) | Box corer | 1.0-30 | 0.001 - 5.0 | 13 | Estero de Urias Lagoon, Mexico (Gulf of California) | 0 - 2247 particles/kg | FI: 86 %, FR: 10 %, PE: 4% n = N.S. | N.S. |  |
|  | Losno-Prado et al. (2023) | Van Veen grab sampler | 1.77 ± 0.47 | 0.05-5.0 | 16 | Los Pantanos de Villa coastal lagoon, Perú | 10.13 ± 3.68 particles/kg | FR: 51.9 %, FL: 25.3 %, FI: 22.8 %  n = N.S. | N.S. |  |
|  | Ruiz-Fernández et al. (2024) | Box corer | 1.5 - 3.3 | 0.1 - 4.3 | 3 | Estero de Urias Lagoon, Mexico (Gulf of California) | 200 - 2278 particle/kg | FI: 80.3 %,FR: 19.7 % n = N.S. | PET: 24.5 %, PE: 1.5 %, PP: 3.3 %, Acrylic: 1.9 %, PA: 0.4 %, RCF: 68.4 % n = 200 |  |
|  | Fernandes et al. (2025) | Van Veen grab sampler | 1.0 - 6.0 | 0.25 - 5.0 | 10 | Santos São Vicente estuary, Brazil (South Atlantic Ocean) | 6150 - 93050 item/kg | FI, FR, FO, FI | PE: 26.7 %, PET: 17.6%, PMMA: 14.7 %, PS: 13.7 %, PP: 8.8 %, PA: 4.8 %, Others: 13.7 % n = N.S. |  |
| Other regions | Wang et al. (2019) | Box corer | 12.0 - 77.0 | 0.05-5.0 | 17 | Southern Yellow Sea, China | 560 - 4205 particles/kg | FI: 83.4 %, GR: 7.5 %, FR: 6.2 %, FL: 2.1 %, SP: 0.6 %, Lines: 0.2 %  n = N.S. | PP: 31.0 %, PES: 24 %, PA: 19 %, PS: 15 %, PET: 6.5 % N.I.: 4.5 %  n = N.S. |  |
|  | Zhang et al. (2019) | Stainless steel grab sampler | 45383 | 0.04 - 4.99 | 28 | Sishili bay, China (Northern Yellow Sea) | 140 - 1873.33 items/kg | FI: 86.4 %, FR: 7.8 %, FL: 5.4 %, PE: 0.4 %  n = N.S. | RA: 58.4 %, PP: 17.8 %, PET: 14.9 %, PE: 5.6 %, PMMA: 1.4 %, PS: 0.9 %, PA: 0.5 %, PU: 0.5%  n = 214 |  |
|  | Sui et al. (2020) | Van Veen grab sampler | N.S. | 0.03 - 5.0 | 8 | Sanggou bay, China (Northern Yellow Sea) | 699-2824 items/kg | GR: 59.0 %, FL: 29.8 %, FI: 10.6 %, PE: 0.6 %  n =N.S. | PE: 47.7 %, PS: 24.5 %, Cell: 10.0 %, PC: 3.8, PP: 14.0 %  n = 5000 |  |
|  | Teng et al. (2020) | Van Veen grab sampler | N.S. | 0.001 - 5.0 | 58 | Laizhou bay, China (Bohai sea) | 193 - 1053 particles/kg | FI: 95.7 %, FL: 3.3 %, FR: 0.5 %, Particle: 0.5 %  n = N.S. | CE: 85.4 %, PET: 8.9 %, PP: 1.4 %, PVA: 1.2 %, PPA: 1.1 %, PA: 0.9 %, PAN: 0.5 %, PE: 0.3 %, PEA: 0.2 %, PVC: 0.1 %  n = N.S. |  |
|  | Zhang et al. (2020) | Box corer | 4601 - 5732 | 0.1 - 5.0 | 15 | Western Pacific Ocean | 0 - 1042 items/kg | FI: 52.5 %, FL: 30.0 %, FR: 17.5 %  n = 40 | Copolymers: 60.0 %, PET: 27.5 %, CE: 10.0 %, PES: 2.5 %  n = 40 |  |
|  | Ye et al. (2023) | Stainless steel grab sampler | 10 | 0.008 - 5.0 | 104 | Liaodong Bay, China (Bohai Sea) | 0 – 86.67 item/kg | FI: 92.9 %, FR: 5.7 %, FL: 0.9 %, PE: 0.5% n = 648 | RA: 87.5 %, PET: 6.8 %, PA: 2.9 %, PP: 2.2%, PE: 0.6 % n = 648 |  |

Supplementary Information references

Astorga A, Montero-Cordero A, Golfin-Duarte G, García-Rojas A, Vega-Bolaños H, Arias-Zumbado F, Solís-Adolio D, Ularte K. 2022. Microplastics found in the World Heritage Site Cocos Island National Park, Costa Rica. Marine and Fishery Sciences 35(2): 430-420 DOI: 10.47193/mafis.3532022010907

Baptista JA, Gaylarde C, Beech I, Bastos AC, da Silva Quaresma V, de Carvalho DG. 2019a. Microplastics and attached microorganisms in sediments of the Vitória bay estuarine system in SE Brazil. Ocean & Coastal Management 169: 247-253 DOI: 10.1016/j.ocecoaman.2018.12.030

Baptista JA, de Carvalho DG, Medeiros K, Drabinski TL, de Melo GV, Silva RCO, Silva DCP, de Sousa Batista L, Dias GTM, da Fonseca EM, dos Santos Filho JR. 2019b. The impact of sediment dumping sites on the concentrations of microplastic in the inner continental shelf of Rio de Janeiro/Brazil. Marine Pollution Bulletin 149: 110558. DOI: 10.1016/j.marpolbul.2019.110558

Borges MM, Dzul R, von Osten JR. 2019. Occurrence and seasonal distribution of microplastics and phthalates in sediments from the urban channel of the Ria and coast of Campeche, Mexico. Science of The Total Environment 672: 97-105 DOI: 10.1016/j.scitotenv.2019.03.472

Cardoso-Mohedano JG, Ruiz-Fernández AC, Sanchez-Cabeza JA, Camacho-Torres SM, Ontivero-Cuadras JF. 2023. Microplastics transport in a low-inflow estuary at the entrance of the Gulf of California. Science of the total Environment 870: 161825 DOI: 10.1016/j.scitotenv.2023.161825

Celis-Hernández O, Ávila E, Rendón-von Osten J, Briceño-Vera A, Borges-Ramírez MM, Gómez-Ponce AM, Capparelli VM. 2023. Environmental risk of microplastics in a Mexican coastal lagoon ecosystem: Anthropogenic inputs and its possible human food risk. Science of the Total Environment 879: 163095 DOI: 10/1016/j.scitotenv.2023.163095

Courtene-Jones W, Maddalene T, James MK, Smith NS, Youngblood K, Jambeck JR, Earthrowl S, Delvalle-Borrero D, Penn E, Thompson RC. 2021. Source, sea and sink-A holistic approach to understanding plastic pollution in the Southern Caribbean. Science of The Total Environment 797: 149098 DOI: 10.1016/j.scitotenv.2021.149098

Fernandes D, Teixeira G, Lima dos Santos J, Ursus N, Rodriguez W, Bereczki A, Barboza M. 2025. Microplastics in Santos São Vicente estuarine – Hotspot in sediments caused by low energy hydrodynamic events in strongly populated areas. Marine Pollution Bulletin 210: 117286 DOI: 10/1016/j.marpolbul.2024.117286

Garcés-Ordóñez O, Saldarriaga-Vélez JF, Espinosa-Díaz LF, Patiño AD, Cusba J, Canals M, Mejía-Esquivia K, Fragozo-Velásquez L, Sáenz-Arias S, Córdoba-Meza T, Thiel M. 2022. Microplastic pollution in water, sediments and commercial fish species from Ciénaga Grande de Santa Marta lagoon complex, Colombian Caribbean. Science of The Total Environment 829: 154643 DOI: 10.1016/j.scitotenv.2022.154643

Jones JS, Porter A, Muñoz-Pérez JP, Alarcón-Ruales D, Galloway TS, Godley BJ, Santillo D, Vagg J, Lewis C. 2021. Plastic contamination of a Galapagos Island (Ecuador) and the relative risks to native marine species. Science of The Total Environment 789: 147704 DOI: 10.1016/j.scitotenv.2021.147704

Losno-Prado AG, Iannacone J. 2023. Characterization of microplastics in water ad sediments in the wetlands Pantanos de Villa, Chorrillos, Lima, Peru. Boletín de Investigaciones Marinas y Costeras 52(1): 185-194 DOI: 10.25268/bimc.invemar.2023.52.1.1155

McEachern K, Alegria H, Kalagher AL, Hansen C, Morrison S, Hastings D. 2019. Microplastics in Tampa Bay, Florida: Abundance and variability in estuarine waters and sediments. Marine Pollution Bulletin 148: 97-106 DOI: 10.1016/j.marpolbul.2019.07.068

Quesada-Rojas M, Enriquez C, Valle-Levinson A. 2021. Natural and anthropogenic effects on microplastic distribution in a hypersaline lagoon. Science of the Total Environment 776: 145803 DOI: 10.1016/j.scitotenv.2021.145803

Ramírez-Álvarez N, Rios Mendoza LM, Macías-Zamora JV, Oregel-Vázquez L, Alvarez-Aguilar A, Hernández-Guzmán FA, Sánchez-Osorio JL, Moore CJ, Silva-Jiménez H, Navarro-Olache LF. 2019. Microplastics: Sources and distribution in surface waters and sediments of Todos Santos Bay, Mexico. Science of The Total Environment 703: 134838 DOI: 10.1016/j.scitotenv.2019.134838

Rendón-von Osten J, Benítez-Torres J, Rojas-González I, Morgado F, Borges-Ramírez M. 2023. Microplastics in sediments from the southern Gulf of Mexico: Abundance, distribution, composition, and adhered pollutants. Science of the Total Environment 873: 162290 DOI: 10.1016/j.scitotenv.2023.162290

Ronda AC, Arias AH, Oliva AL, Marcovecchio JE. 2019. Synthetic microfibers in marine sediments and surface seawater from the Argentinean continental shelf and a Marine Protected Area. Marine Pollution Bulletin 149: 110618 DOI: 10.1016/j.marpolbul.2019.110618

Ruiz-Fernández AC, Pérez-Bernal LH, Sanchez-Cabeza JA, Valencia-Castañeda G, Ontiveros-Cuadras JF, Alonso-Hernández MA. 2024. Accelerating microplastic contamination in 210Pb dated sediment cores from an urbanized coastal lagoon (NW Mexico) since the 1900s. Science of the total Environment 951: 1756613 DOI: 10/1016/j.scitotenv.2024.175613

Sánchez-Campos M, Ponce-Vélez G, Sanvicente-Añorve L, Alatorre-Mendieta M. 2024. Microplastic contamination in three environmental compartments of a coastal lagoon in the southern Gulf of Mexico. Environmental Monitoring and Assessment 196: 1012 DOI: 10/1007/s10661-024-13156-2

Sui Q, Zhang L, Xia B, Chen B, Sun X, Zhu L, Wang R, Qu K. 2020. Spatiotemporal distribution, source identification and inventory of microplastics in surface sediments from Sanggou Bay, China. Science of The Total Environment 723: 138064 DOI: 10.1016/j.scitotenv.2020.138064

Teng J, Zhao J, Zhang C, Cheng B, Koelmans AA, Wu D, Gao M, Sun X, Liu Y, Wang Q. 2020. A systems analysis of microplastic pollution in Laizhou Bay, China. Science of The Total Environment 745: 140815 DOI: 10.1016/j.scitotenv.2020.140815

Vásquez-Molano D, Molina A, Duque G. 2021. Distribución espacial y aumento a través del tiempo de microplásticos en sedimentos de la bahía de Buenaventura, Pacífico colombiano. Boletín de Investigaciones Marinas y Costeras 50 (1): 27-42 DOI: 10.25268/bimc.invemar.2021.50.1.1021

Wang F, Wong CS, Chen D, Lu X, Wang F, Zeng EY. 2018. Interaction of toxic chemicals with microplastics: a critical review. Water Research 139: 208-219 DOI: 10.1016/j.watres.2018.04.003

Ye Y, Zhang A, Teng J, Yang X, Yuan X, Wang Q, Zhao J, Zhang B, Zhang T, Chen X, Guan T, Ma X. 2023. Pollution characteristics and ecological risk of microplastic in sediments of Liaodong Bay from the northern Bohai Sea in China. Marine Pollution Bulletin 187: 114505 DOI: 10.1016/j.marpolbul.2022.114505

Zhang B, Wu D, Yang X, Teng J, Liu Y, Zhang C, Zhao J, Yin X, You L, Liu Y, Wang Q. 2019. Microplastic pollution in the surface sediments collected from Sishili bay, North Yellow Sea, China. Marine Pollution Bulletin 141: 9-15 DOI: 10.1016/j.marpolbul.2019.02.021

Zhang D, Liu X, Huang W, Li J, Wang C, Zhang D, Zhang C. 2020. Microplastic pollution in deep-sea sediments and organisms of the Western Pacific Ocean. Environmental Pollution 259: 113948 DOI: 10.1016/j.envpol.2020.113948
